# Supplementary material for: AI-based coral species discrimination: A case study of the Siderastrea Atlantic Complex
Source: PLoS One. 2024 Dec 11;19(12):e0312494. doi: 10.1371/journal.pone.0312494 (PMC11634003; doi:10.1371/journal.pone.0312494)
Supplement: S1 File — (PDF) [file pone.0312494.s001.pdf]

Supplementary Information for

AI-based coral species discrimination: a case study of the *Siderastrea* Atlantic Complex

Marcos Barbeitos, Flavio Pérez, Julián Olaya-Restrepo, Ana Paula Winter, João Florindo and Estevão Laureano

Corresponding Authors
Marcos Soares Barbeitos msbarbeitos@gmail.com,
João Batista Florindo jbflorindo@ime.unicamp.br,
Estevão Esmi Laureano eelaureano@gmail.com

This PDF file includes:

Supplementary text
Figs. S1 to S14
Tables S1 to S7
References for SI reference citations

Supporting Information Text

Brief review of *Siderastrea* taxonomy and its issues

The genus was first described in the eighteenth century by Pallas (1). It was called *Madrepora* and the publication makes reference to *Madrepora radians* from the "*Mare americanum*" and *Madrepora stellaris*, located in the Indian Ocean. Milne-Edwards (2), through *S. galaxea*, erected the genus *Siderastrea*, described as convex, encrusting colonial corals of dense tissue, fine corallites joined by their walls, deep pits and papilose, poorly developed columella, with thin, closely spaced, denticulate septa. Edwards (3) described *Astrea radians* (transferred from *Madrepora*) as having "the great diagonal of the chalices of 3.0 or 4.0 mm; and its depth, 2.0 or a little more. (...) Columella formed by one or two very compact, visible tubers, 3 complete cycles and, in general, a variable number of fourth cycle partitions that are unusual in many systems". He also described *Astrea siderea*, "undeveloped columella, reduced to two or three small papillae (. . .) large diagonal of the chalices of 4.0 to 5.0 mm, its depth 2.0". In 1830, de Blainville (4) transferred *Astrea* to *Siderastrea*.

In the beginning of the twentieth century, Duerden (5), in his work on *S. radians*, disagreed with Edwards (3) and Verrill (6), who argued that it is common to consider that all internal cycles are complete in hexameric arrangement and all absent septa belong to the last cycle. On the other hand, referring to the development of the septa, Duerden (5) states that "it is clear that such cyclic plans do not express the true ordinal or morphological relationships of the septa", considering that the last and the penultimate cycle vary in the same degree, that is, septa may be absent in any cycle.

Gregory (7) considered *S. stellata* a synonym of *S. siderea*. However, Vaughan (8) argued that *S. stellata* is a very distinct species, with "deformed calyces, smaller diameter of 2.0 to 3.0 mm, length of up to 6.5 mm or more and finely papillary columella". *S. siderea* presents "deep columellar fossa, quite narrow in the background; calyces 3.0 to 5.0 mm in diameter, with tertiary septa which normally fuse to the secondary ones distinctly behind the columella; 6 to 8 septal teeth at 1 mm" and *S. radians* has calyces rarely larger than 4 mm in diameter, columella composed of 1 to 3 fused papillae. He also reports that *S. stellata* "resembles *S. radians* by having the fourth cycle of incomplete septa, outer banks of flattened septa and very steep internal margins, and a deep columellar fossa. It differs (...) by having deeper calyces, which may be meandering, by its more crudely toothed septa, and by its much less developed, finely papillary columella (...) in addition, *S. siderea* possesses larger masses" (colonies). Therefore, *S. stellata* can not be synonym of *S. siderea*, according to the author. On the other hand, Budd & Guzman (9) and Reyes et al. (10) reported chalice diameters of 2.5 to 3.5 mm, whereas diameters of *S. siderea* ranged from 3.0 to 5.0 mm. *S radians* and *S. stellata* undergo intratentacular budding whereas *S. siderea* does not (Neves pers. comm.). Vaughan (11) remarked about *Siderastrea radians*, still called *Madrepora radians*, that: "Although the main mode of colony formation is by extratentacular budding, the intratentacular mono-stomodaeal mode is not uncommon in the species living in the Brazilian and Gulf of Guinea reef areas".

Siderastrea glynni, described by Budd & Guzman (9), would be the only species of the genus to occur in the Eastern Tropical Pacific. However, molecular data from the ITS haplotypes demonstrated that almost all sequences were identical to those of *S. siderea* (12). In addition, Lajeunesse et al. (13) verified the presence of two *Symbiodinium* species in the *S. glynni* samples that are also found in Atlantic *S. siderea*. Glynn et al. (14) presented evidence that *S. siderea* was inadvertently introduced from the Caribbean into the Pacific Ocean in 1982, and those would be the same fragments found by Guzman in 1992. Thus, the authors state that *S. glynni* should be synonymized to *S. siderea*, and that the slightly different morphological characteristics of the skeleton were possibly due to phenotypic plasticity.

The debate about *Siderastrea glynni* illustates how important the biogeography of the species within this genus is when it comes to their taxonomy, in part because diagnostic characters are so variable, as reviewed above. *S. stellata* was, until recently, regarded as a species endemic to Brazil, ranging from Maranhão (00° S, 44° W) to Rio de Janeiro state (23° S, 42° W)(15–19) and considered the main reef-building organism in some sites such as the Atol das Rocas (20). However, García et al. (21), using integrated morphological and molecular approaches, recorded the presence of *S. stellata* in the Gulf of Mexico, although the molecular phylogenies using transcribed internal spacers (ITS1 and ITS2) did not recover reciprocally monophyletic species. On the other hand, *S. siderea* has been historically reported as occurring in the northern hemisphere only, but Neves et al. (22) reported for the first time the occurrence of *S. siderea* in the Abrolhos bank, off the coast of the state of Bahia, based on the morphological analysis of a single corallum (4.0 x 3.0 cm), collected during the Hartt expedition (1874 - 1876), and deposited at the NMNH, Smithsonian Institution, Washington (DC). The characteristics considered by the authors were the diameter of the corallite (3.5 mm to 4.0 mm), the number of septa (40-64, with fourth cycle ranging from 16-24 septa), septal arrangement (distributed regularly in four and sometimes five cycles), type and number of dentition of the primary septum (thin, 0.1 mm wide and 2.0 mm long, with 10 to 13 dentitions), diameter (0.8 mm) and depth of the columella (2.0 - 3.0 mm), number of papillae (3-6) and number of synapticular rings (4). The aspect of the corallum (massive growth form, an incrustation base and a distinct oval surface) was also considered a diagnostic feature.

Santos et al. (23) analyzed the skeletal morphology of *S. stellata*, comparing it with *S. siderea* and *S. radians*, in order to evaluate the possible latitudinal differences in the populations of *Siderastrea*. They employed four distinct characters: corallite diameter, columellar distance, total number of septa by corallite and number of columella per cm^2. *S. stellata* samples from the neighboring states of Paraíba and Pernambuco (Brazil) were phenetically closer to *S. siderea* and *S. radians* from Panama, respectively, than to each other. This finding predates the work by Neves et al. (22) and supports the presence of *S. siderea* in Brazil. Menezes et al. (19) reviewed works on the genre as the one by Werner (24) who considered *S. stellata* and *S. radians* as synonyms, based on morphometric analysis. However, all synonyms were refuted by genetic data, since there is evidence of reproductive isolation and the presence of private alleles were found in both *S. stellata* and *S. radians*

(19, 25, 26). Additionally, they analyzed the characters of the corallites and evaluated the magnitude of variation within and between colonies of *S. stellata* and *S. radians*. They distinguished among three different zones on the colony surface (upper, intermediate and border), selecting six quantitative parameters: diameter of corallite and columella (based on the average of two larger diameters), columellar diameter, number of septa, thickness of theca (which includes the outer and all synaptic walls), depth of the columellar fossa and the mean distance between adjacent corallites. In both studies, the authors reported significant morphological variation between *S. stellata* and *S. radians*, considering the number of septa as the most important diagnostic morphological character.

The evaluation of the genetic structure of populations with the use of isoenzymes distributed along 2,000 km of the Brazilian coast confirmed the occurrence of *S. radians* in Brazil, although Caribbean samples were not included in the sampling design (26). The first phylogenetic analysis at the species level confirmed the divergence between *S. stellata*, *S. radians* and *S. siderea* in a phylogenetic study based on ITS2 (12). Nunes et al. (27) found shared haplotypes between *S. radians* and *S. siderea*, and suggested that *S. stellata* would be a hybrid of both species.

The mode of reproduction also varies among *Siderastrea* species. *S. stellata* and *S. radians* are gonochoric (i.e. have separate sexes), have internal fertilization and brood their embryos(19, 25, 26, 28), whereas *S. siderea*, although also gonochoric, releases the gametes in the water for external fertilization (broadcast spawner - Neves pers. comm.). The type of reproduction is related with the dispersion potential. The incubated planulas are released at an advanced stage of development and ready for settlement (26, 29). Laboratory experiments have shown that *S. radians* planula remain competent between 24 and 48 h, while *S. stellata's* are unable to initiate a metamorphosis within 72 h (25, 26). Neves & da Silveira (25) observed the fusion of larvae of the same colony in *S. stellata* which, according to the authors, could lead to an increase in recruitment success. Although *S. radians* apparently have a lower dispersion capacity when compared to *S. stellata*, the species has already been recorded from Rio Grande do Norte to Espírito Santo states, while *S. stellata* occurs from Maranhão to Rio de Janeiro and (19, 26). In addition, *S. stellata* presents biased sex-ratio towards males and annual reproductive cycle, planulates between January and February (30, 31). All of these characteristics suggest that there is a greater probability of reproductive isolation between *S. siderea* and the other two species, that is, the possibility of hybridization suggested by Nunes et al. (27) is quite unlikely.

Brief description of Fuzzy ANNs

Artificial neural networks (ANNs) are efficient classifiers inspired in biological nervous systems. These networks are made up of artificial neurons, computational objects that hold real numbers and are arrayed in layers connected by numeric constants called weights. The input layer has as many artificial neurons as entries in the input vectors. The output layer has as many neurons as the pre-defined categories (two or three species, in our case). Weights are estimated parameters that minimize classification error in cross-validation experiments, collectively known as ”network training” because, as weights are optimized, the network ”learns” and ”memorizes” how to correctly associate input-ouput pairs. Once training is finished, the value in output neurons is given by the weighted sum of the input values or, algebraically speaking, by a linear transformation of input into output. However, between input and output sits one or more hidden (intermediate) layers that are also made up of artificial neurons. These neurons will only ”spike” (i.e. contribute information to the output) if the weighted sum of the values in the previous (input or hidden) layer exceeds a certain threshold. This threshold is set by a nonlinear activation function (e.g. logistic) that may, for instance, transform negative values into 0 (inhibition) and positive values into 1 (excitation). This binary activation process mimics synaptic transmission and makes it possible to approximate continuous nonlinear functions to any desired degree of precision, simply by adding more hidden layers to the network. Therefore, unlike other techniques (e.g. Discriminant Analysis), ANNs are also able to discriminate among classes that are not linearly separable(32).

One can further enhance the efficiency of the network by implementing a competitive layer, in which groups of neurons compete for the right to spike. As network training proceeds, such groups specialize in subsets of the input data and remove redundancy in internal feature representation, because only one neuron per group will spike at any given time. For example, building a competitive layer into an ANN designed to classify cat and dog images may generate a group of neurons specialized in slit-like pupils, a telltale feature of a cat.

Fuzzy set theory was introduced in 1965 by Lofti Zadeh(33) and aims to provide a mathematical treatment of objects or concepts with imprecise boundaries as described in natural language (e.g. very vs. mildly dirty, high vs. low temperatures, fresh vs. rotten tomato, etc.)(34). In a fuzzy neural network, weights, input and output vectors are treated as fuzzy sets(35) and input-output associations are established by equivalent measures, functions that evaluate the degree of similarity between such sets(36, 37). They have been successfully applied to several benchmark datasets available on the internet(38–41) and other real problems such as vision based self-localization of a mobile robot(39) and identification of human speakers(38).

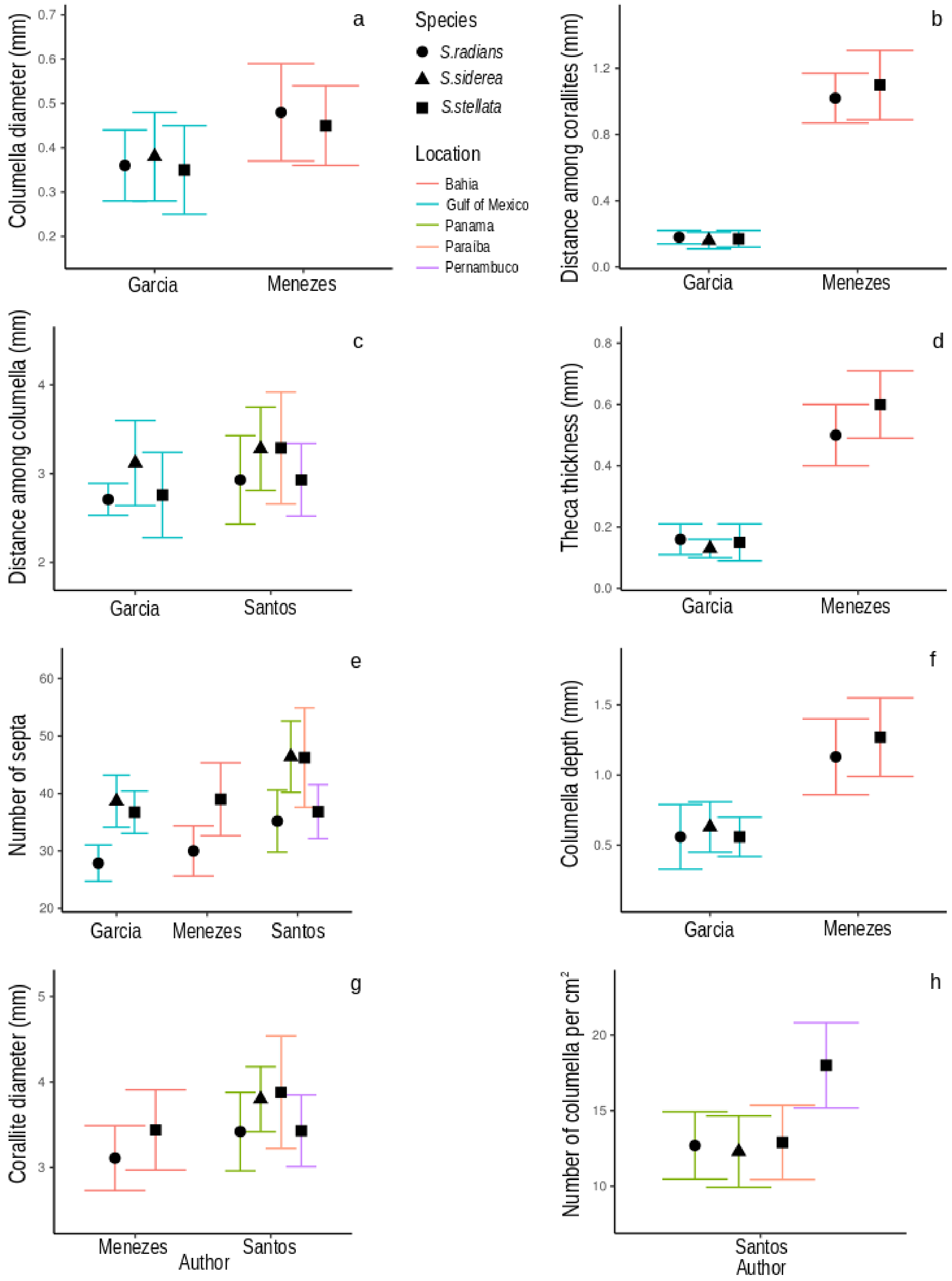

Fig. S1. Averages and confidence intervals, calculated as $\pm$ 1.96 x standard deviations, from three published papers(19, 21, 23) reporting those statistics for 8 types of characters sampled from specimens collected in Brazil, Panama (Bocas del Toro $\approx$ 10°N, 82°W) and Mexico (Vera Cruz $\approx$ 19°N, 96°W), belonging to the three species that make up the *Siderastrea* Complex of the Atlantic. Species are identified by the symbols representing averages and locations are color coded by lines, according to the legend. Papers are identified by the last name of the first authore. Bahia = State of Bahia, Brazil ($\approx$ 12°S, 38°W); Paraíba = State of Paraíba, Brazil ($\approx$ 07°S, 35°W); Pernambuco = State of Pernambuco State, Brazil ($\approx$ 08°S, 35°W).

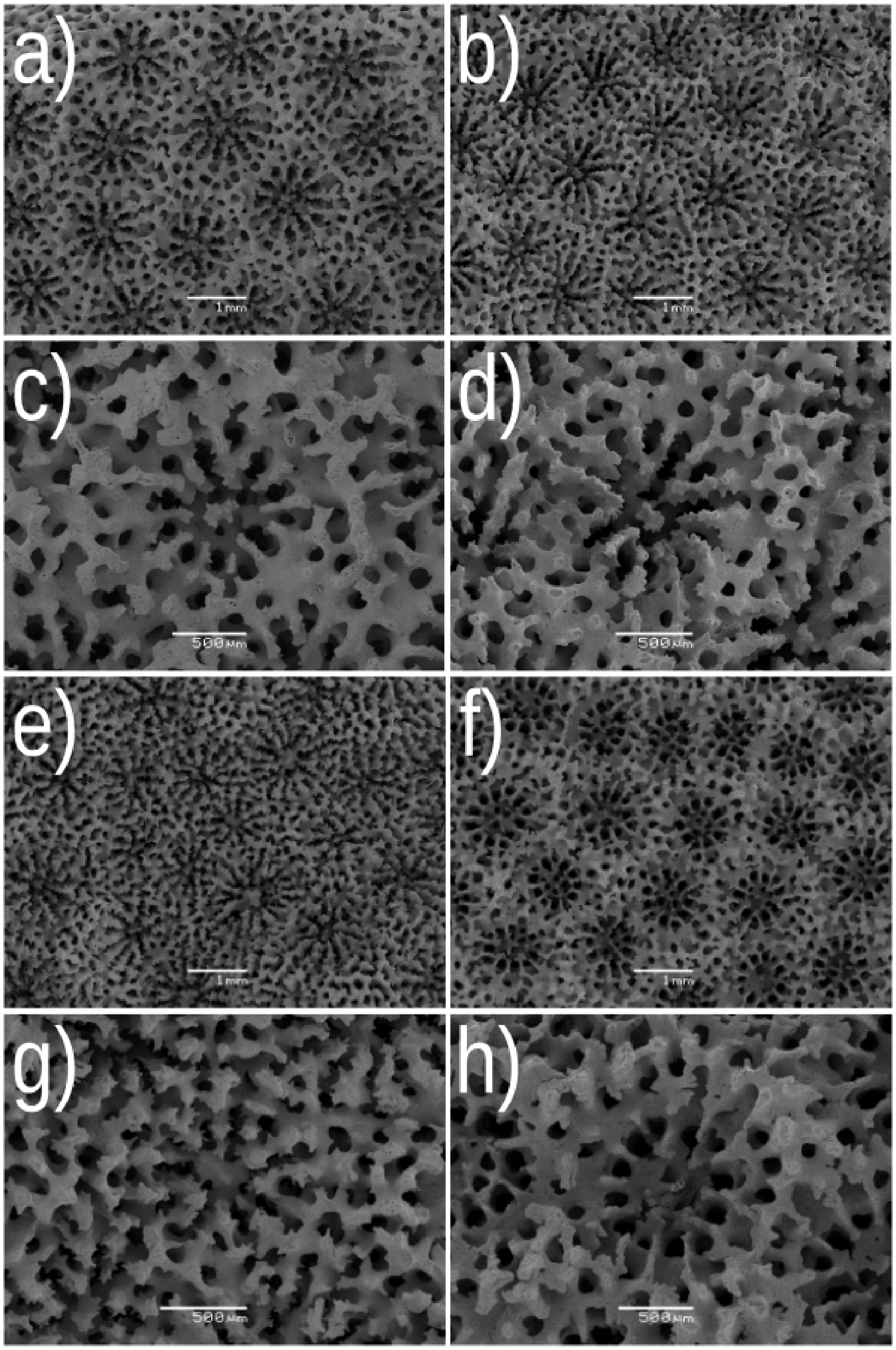

Fig. S2. SEM micrographs illustrating variation in *Porites* spp. corallite structure. a) Colony scale micrograph - *P. astreoides*, voucher 045, Abrolhos Archipelago (4m); b) Colony scale micrograph - *P. branneri*, voucher 080, Abrolhos Archipelago (5m); c) Corallite scale micrograph - *P. astreoides*, voucher 045, Abrolhos Archipelago (4m); (d) Corallite scale micrograph - *P. branneri*, voucher 080, Abrolhos Archipelago (5m); e) Colony scale micrograph - *P. astreoides*, voucher 228, Tamandaré (5m); f) Colony scale micrograph - *P. astreoides*, voucher 263, Tamandaré (5m); g) Corallite scale micrograph - *P. astreoides*, voucher 228, Tamandaré (5m); f) Corallite scale micrograph - *P. astreoides*, voucher 263, Tamandaré (5m)

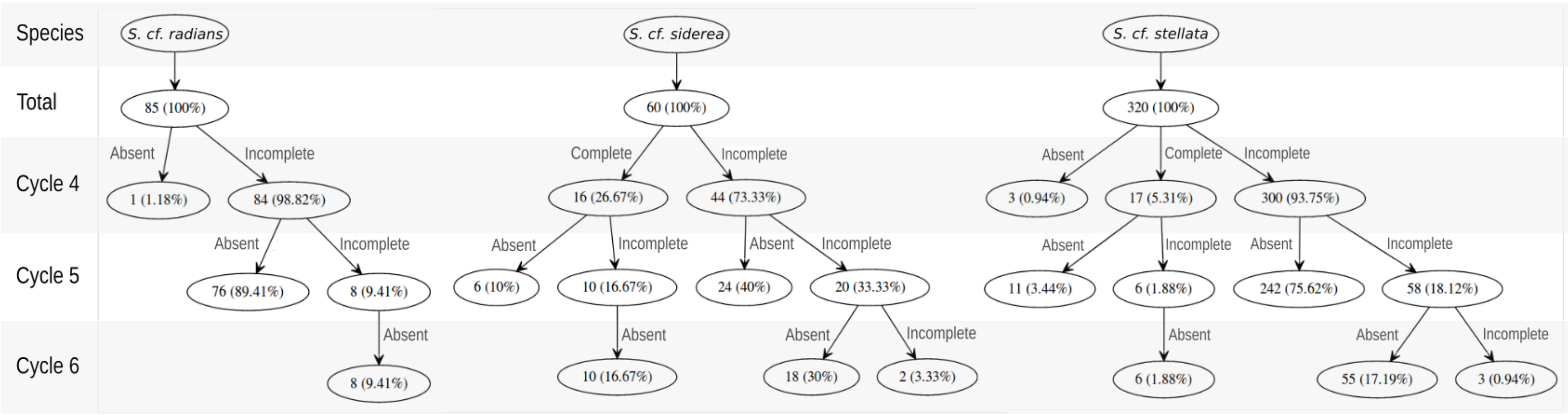

Fig. S3. Breakdown of individual corallites according to presence/absence and completeness of cycles with orders greater than 3.

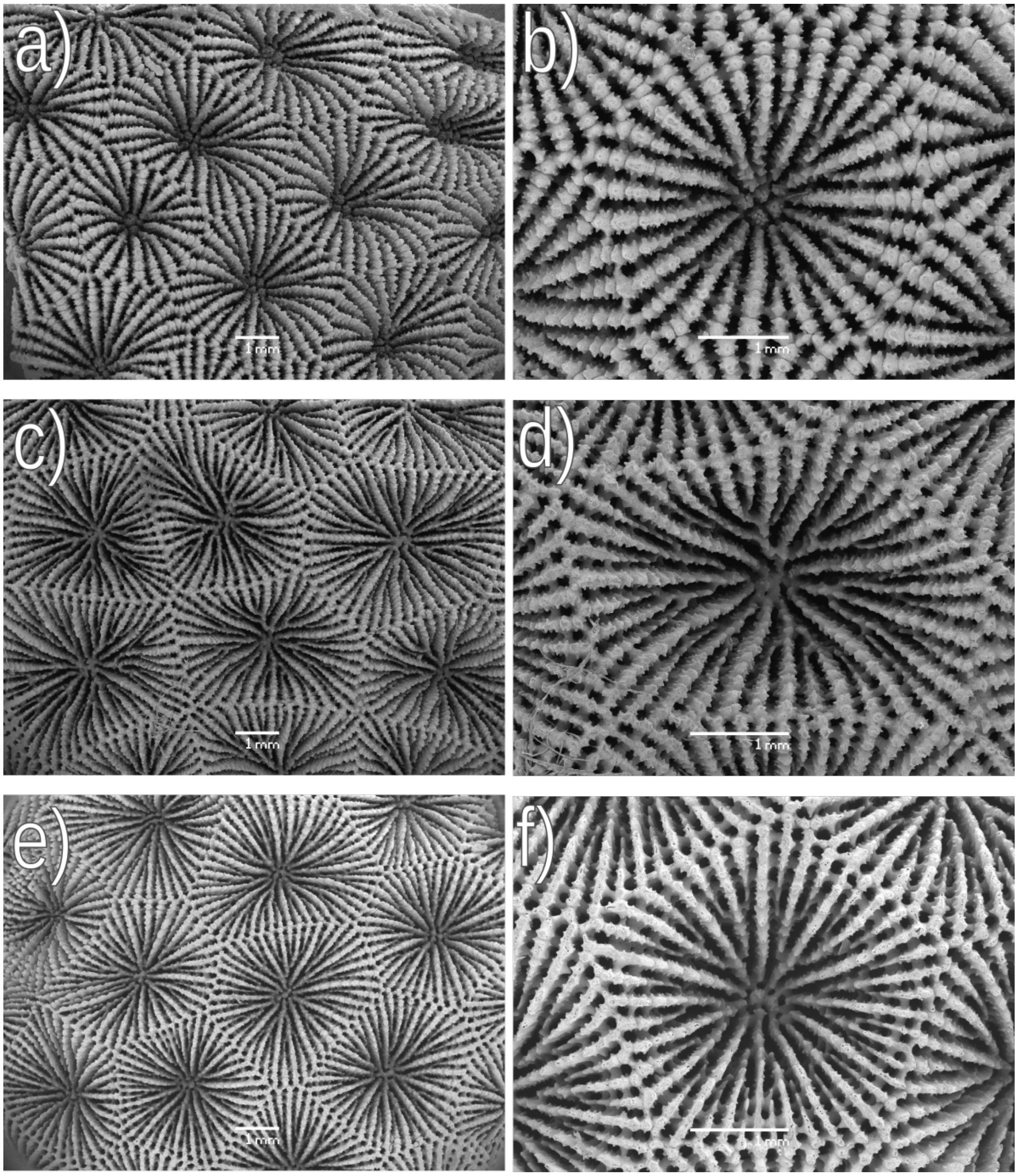

Fig. S4. SEM micrographs illustrating variation in *Siderastreae* spp. corallite structure. a) Colony scale micrograph - *S. cf. radians*, voucher 113 b) Corallite scale micrograph - *S. cf. radians*, voucher 113 c) Colony scale micrograph - *S. cf. siderea*, voucher 027 d) Corallite scale micrograph - *S. cf. siderea*, voucher 027 e) Colony scale micrograph - *S. Stellata*, voucher 023 f) Corallite scale micrograph - *S. cf. stellata*, voucher 023

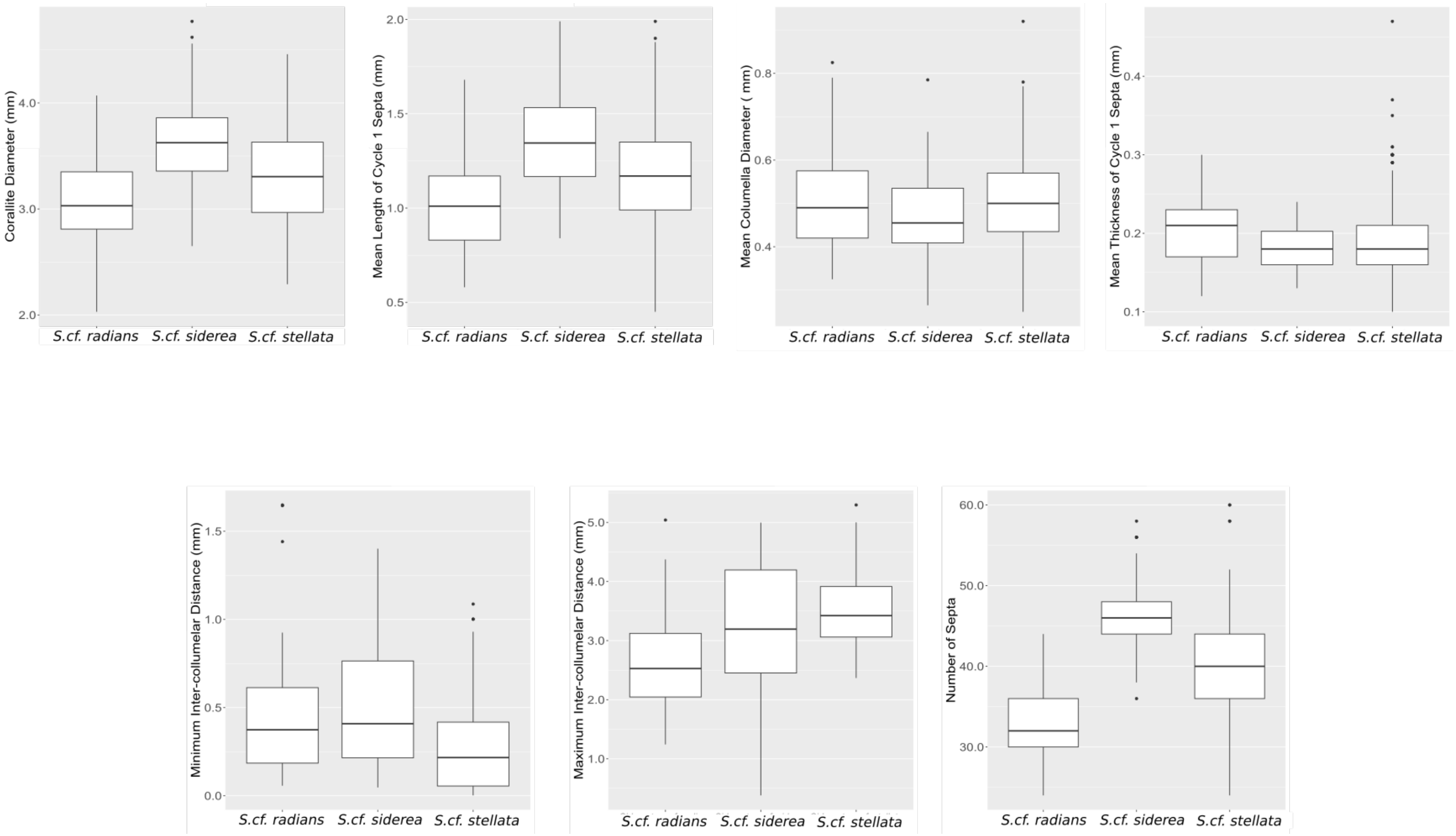

Fig. S5. Boxplots of continuous morphometric characters sampled in this study. Boxes are interquartile ranges, center lines are medians, vertical bars are non-outlier ranges and outliers are represented as dots.

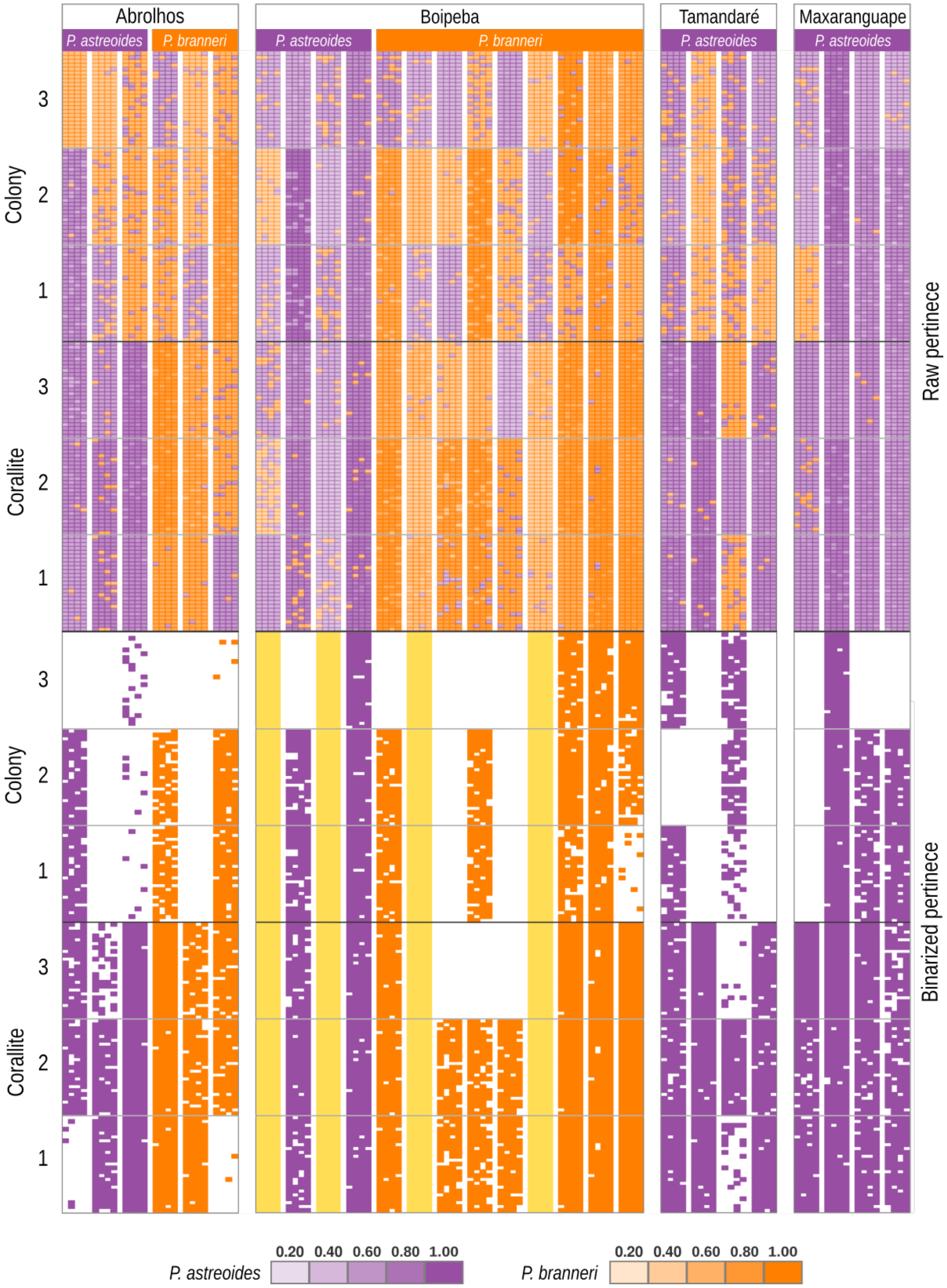

Fig. S6. Mosaic plot showing results for all (n = 100) 5-fold cross-validation experiments of Θ-FAM applied to *Porites* spp. CLBP. Each tile corresponds to one experimental trial for one image. Rectangles aggregate 100 trials for each image and numbers on the right of the panels identify the image number and the scale at which the micrographs were taken. Columns on each panel correspond to the 6 images taken of each colony and are arranged geographically along the left-right direction (identified at the top) from the southernmost (Abrolhos Archipelago, BA) to the northernmost collection site (Maxaranguape, RN - see map in Fig. 1 of the main text). Tiles in the upper panel are color-coded (see legend at the bottom) according to the maximum pertinence value obtained for one of the two possible species. Ties were always resolved as false negatives, so that the mosaic represents the "worst case scenario". Tiles in the bottom panel are colored if true positives i.e., maximum pertinence of the corresponding trial is equal to or larger than binarization threshold for that trial and matches the correct species, identified in the horizontal bars at the top of the plot. Columns blocked in yellow in the lower panel correspond to colonies whose images failed to produce true positives in every cross-validation experiment.

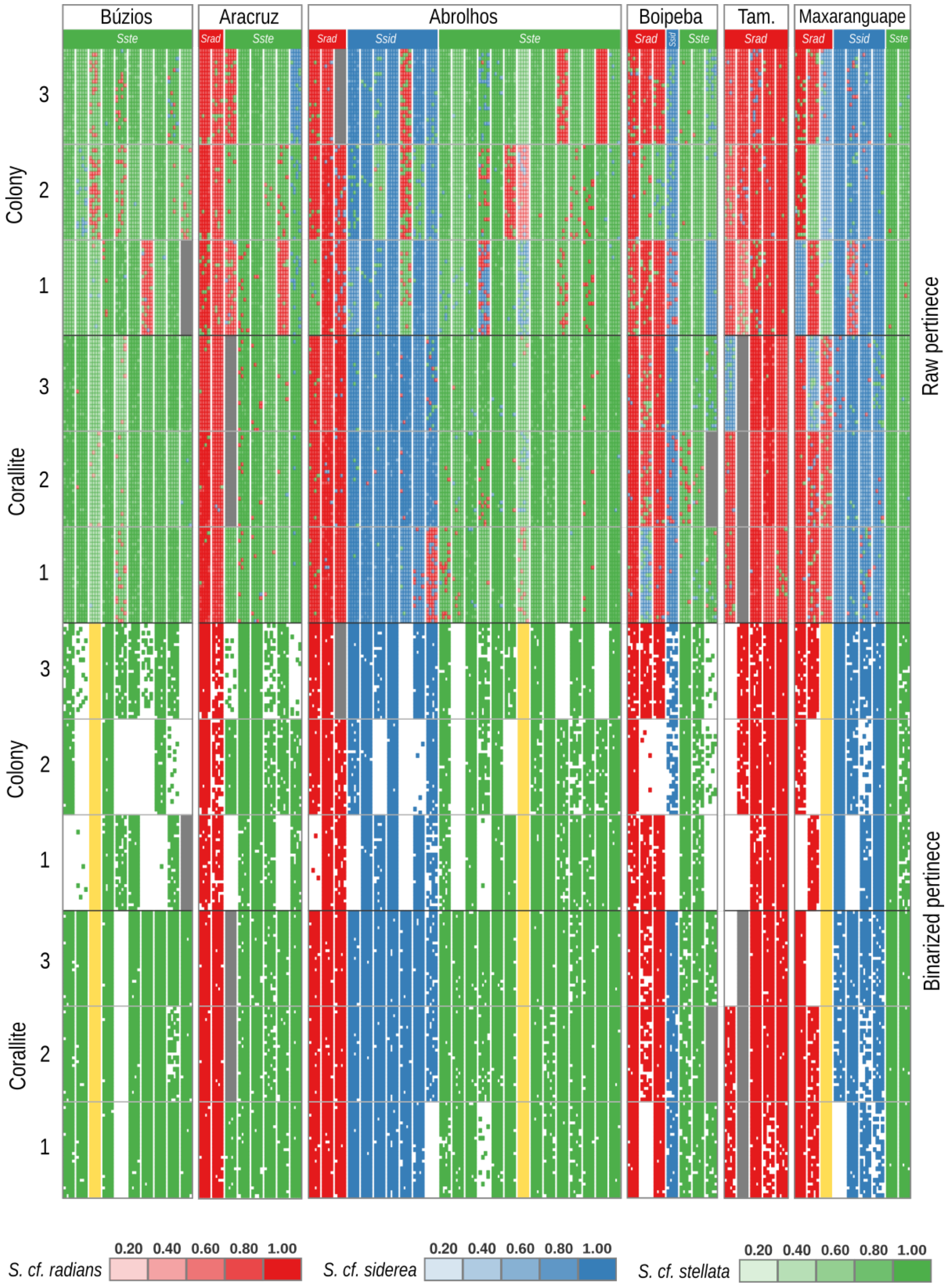

Fig. S7. Mosaic plot showing results for all (n = 100) 5-fold cross-validation experiments of Θ-FAM applied to *Siderastrea* spp. CLBP. Each tile corresponds to one experimental trial for one image. Rectangles aggregate 100 trials for each image and numbers on the right of the panels identify the image number and the scale at which the micrographs were taken. Columns on each panel correspond to the 6 images taken of each colony and are arranged geographically along the left-right direction (identified at the top) from the southernmost (Armação dos Búzios, RJ) to the northernmost collection site (Maxaranguape, RN - see map in Fig. 1 of the main text). Tiles in the upper panel are color-coded (see legend at the bottom) according to the maximum pertinence value obtained for one of the three possible species. Ties were always resolved as false negatives, so that the mosaic represents the "worst case scenario". Tiles in the bottom panel are colored if true positives i.e., maximum pertinence of the corresponding trial is equal to or larger than binarization threshold for that trial and matches the correct species, identified in the horizontal bars at the top of the plot. Images blocked in gray were discarded because the photographed portion of the colony was partially eroded. Columns blocked in yellow in the lower panel correspond to colonies whose images failed to produce true positives in every cross-validation experiment. Tam. = Tamandaré.

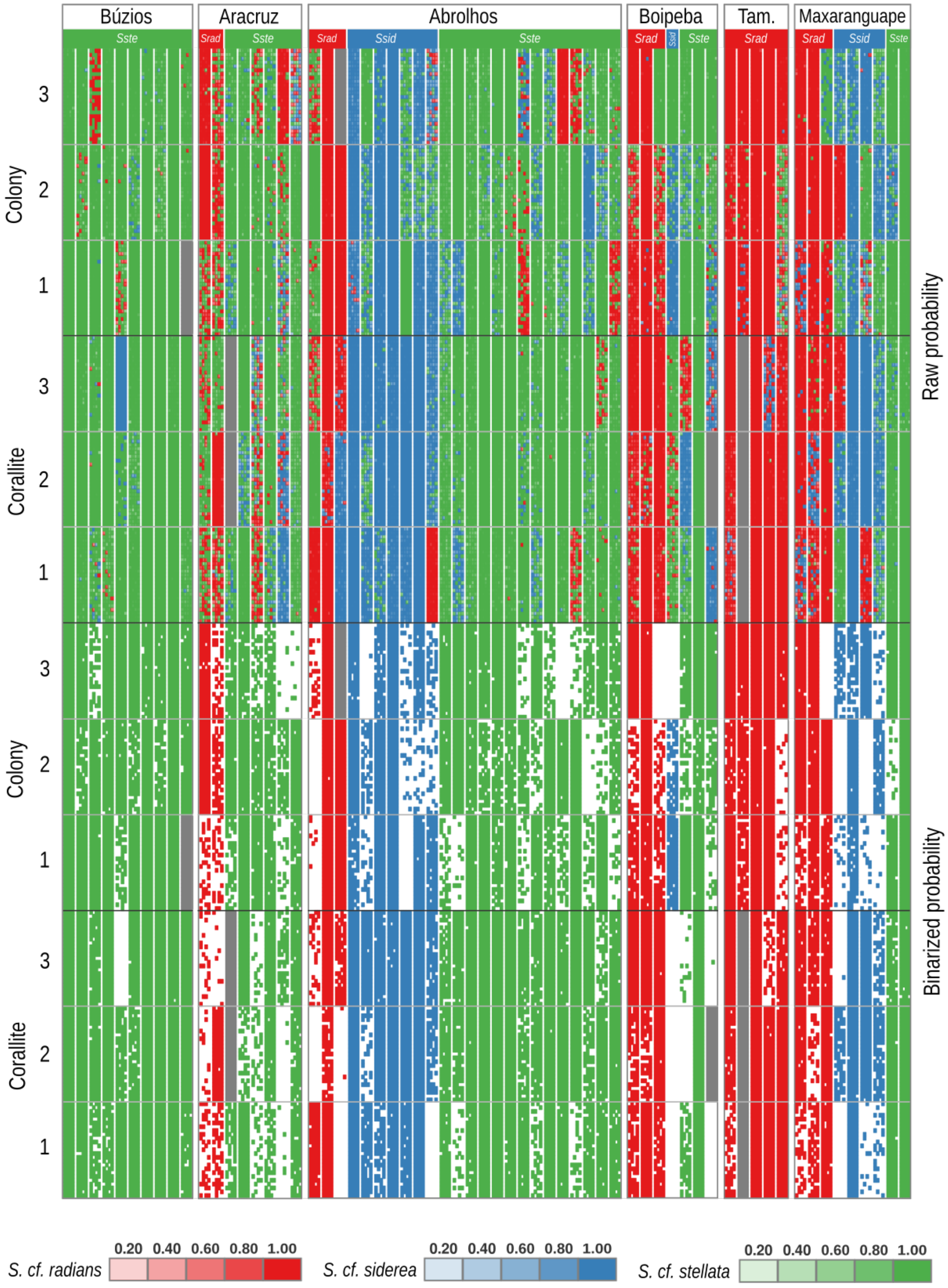

Fig. S8. Mosaic plot showing results for all (n = 100) 5-fold cross-validation experiments of DPCA applied to *Siderastrea* spp. CLBP. Each tile corresponds to one experimental trial for one image. Rectangles aggregate 100 trials for each image and numbers on the right of the panels identify the image number and the scale at which the micrographs were taken. Columns on each panel correspond to the 6 images taken of each colony and are arranged geographically along the left-right direction (identified at the top) from the southernmost (Armação dos Búzios, RJ) to the northernmost collection site (Maxaranguape, RN - see map in Fig. 1 of the main text). Tiles in the upper panel are color-coded (see legend at the bottom) according to the maximum pertinence value obtained for one of the three possible species. Ties were always resolved as false negatives, so that the mosaic represents the "worst case scenario". Tiles in the bottom panel are colored if true positives i.e., maximum pertinence of the corresponding trial is equal to or larger than binarization threshold for that trial and matches the correct species, identified in the horizontal bars at the top of the plot. Rectangles blocked in gray correspond to images discarded *a posteriori* because of damage inflicted on the colony surface. Tam. = Tamandaré.

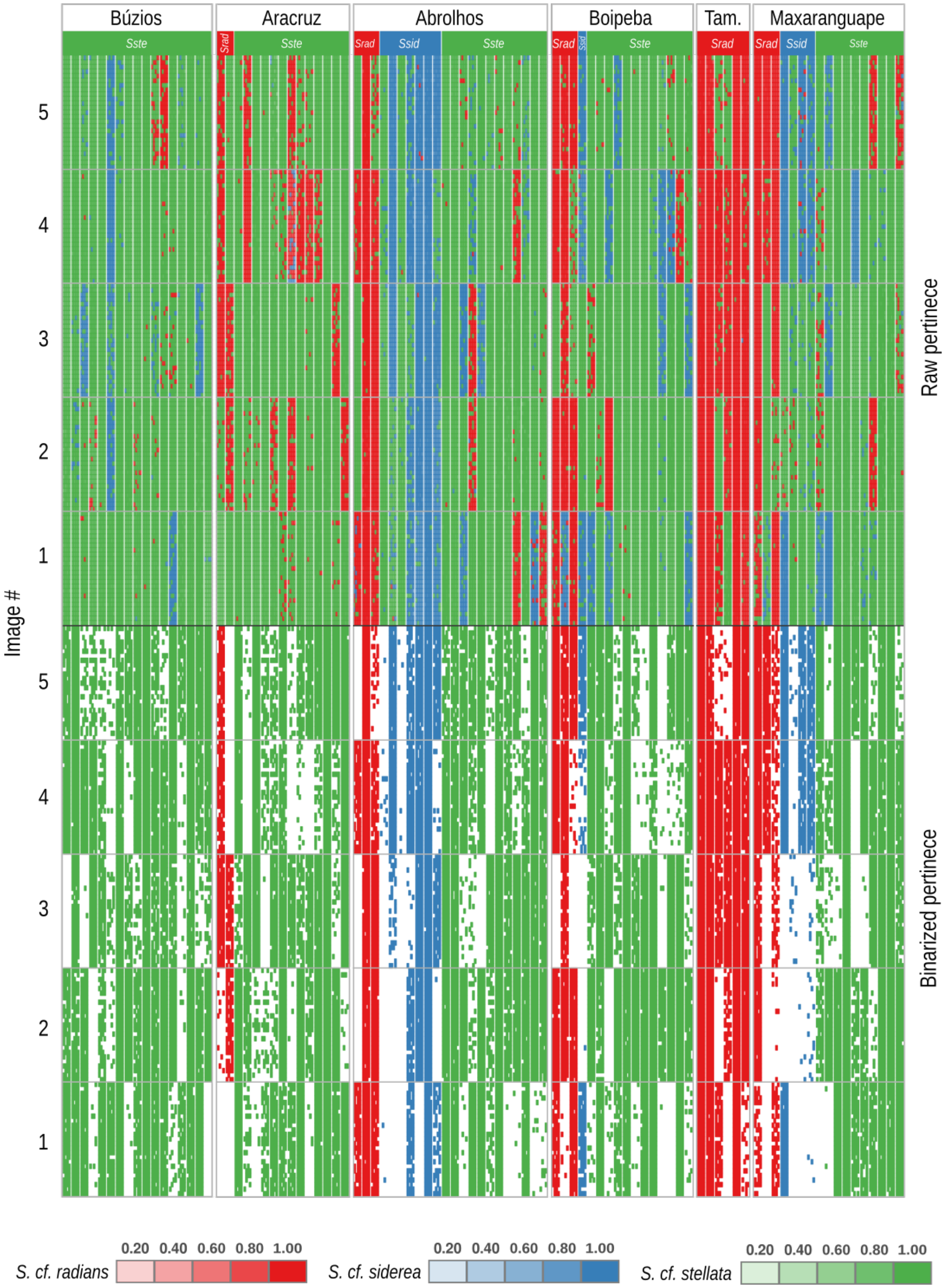

Fig. S9. Mosaic plot showing results for all (n = 100) 5-fold cross-validation experiments of Θ-FAM applied to *Siderastrea* spp. morphometric traits. Each tile corresponds to one experimental trial for one set of traits collected from each image. Rectangles aggregate 100 trials for each set and numbers on the right of the panels identify the image. Columns on each panel correspond to the 5 images analyzed for each colony and are arranged geographically along the left-right direction (identified at the top) from the southernmost (Armação dos Búzios, RJ) to the northernmost collection site (Maxaranguape, RN - see map in Fig. 1 of the main text). Tiles in the upper panel are color-coded (see legend at the bottom) according to the maximum pertinence value obtained for one of the three possible species. Ties were always resolved as false negatives, so that the mosaic represents the "worst case scenario". Tiles in the bottom panel are colored if true positives i.e., maximum pertinence of the corresponding trial is equal to or larger than binarization threshold for that trial and matches the correct species, identified in the horizontal bars at the top of the plot. Tam. = Tamandaré.

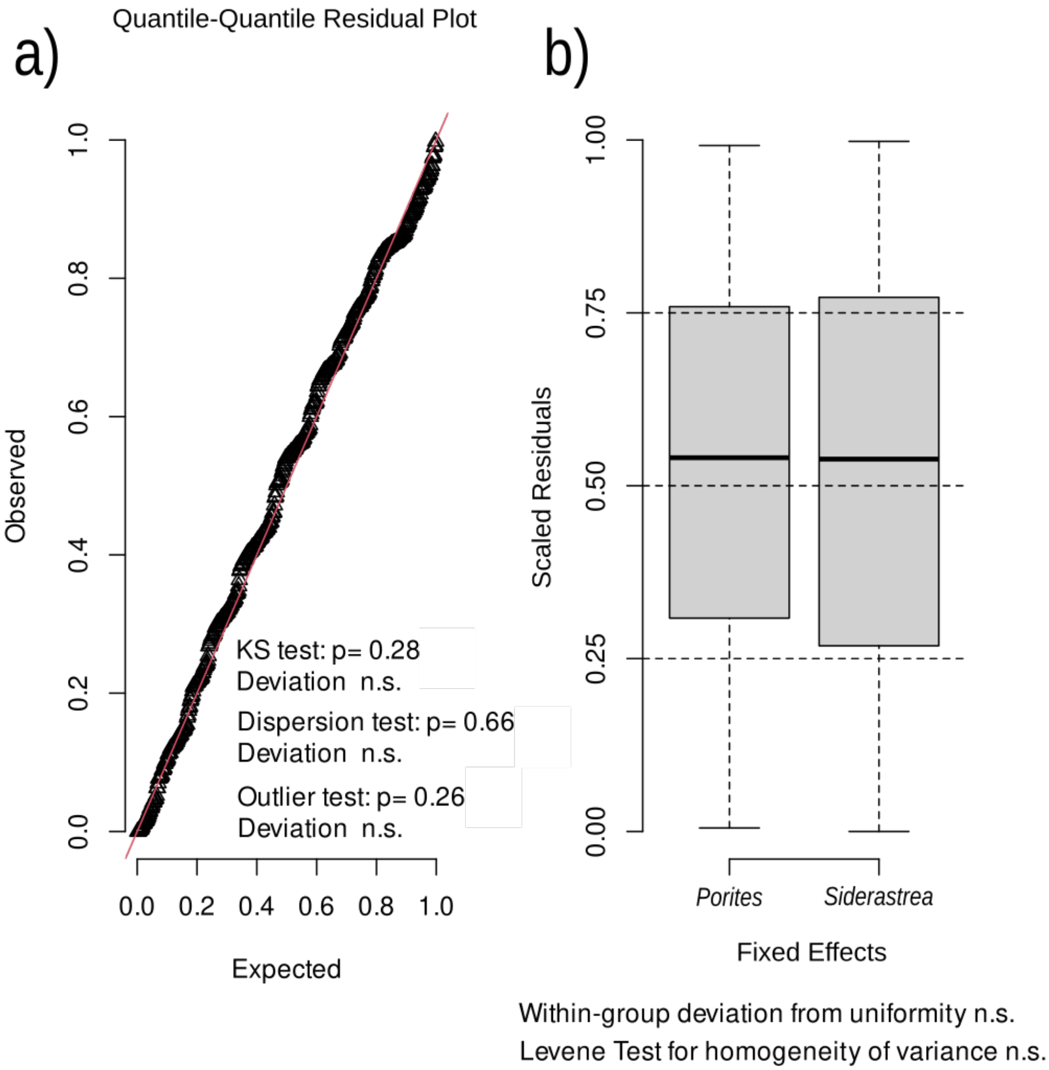

Fig. S10. Residual analysis of GLMM comparing the performance of Θ-FAM classification of CLBP from *Porites* spp. and *Siderastrea* spp. using the R package DHARMa(42). a) Quantile-quantile (QQ) plots of observed and expected residuals distributions, computed from 1000 simulations. DHARMa uses the fitted model as a generative function to simulate expected residuals for the actual dataset. If the observed and simulated distributions agree, quantiles will follow the reference line, indicating that the adjusted model shows good fit to the empirical data. Discordance between distributions is also evaluated using Kolmogorov-Smirnov (KS), overdispersion and outlier tests, which were all non-significant. b) Uniformity and variance homogeneity test of re-scaled residuals within and among predictors. If half of the cumulative density computed for the simulated residuals is above the actual residual value for each observation and half of the density is below the actual residual, the re-scaled observed residual is 0.5. If the re-scaled residual is greater than 0.5, simulated residuals are actually smaller than expected and the opposite is true if the re-scaled residual is lower than 0.5. Hence, all values of the cumulative distribution (i.e. re-scaled residuals) should have the same probability of being sampled when the empirical data was "created" by the same generative model used in the simulations. This means a flat (uniform) distribution of re-scaled residuals within treatment levels, regardless of the fitted model structure. Uniformity was not rejected and variance is homogeneous between fixed-effect levels.

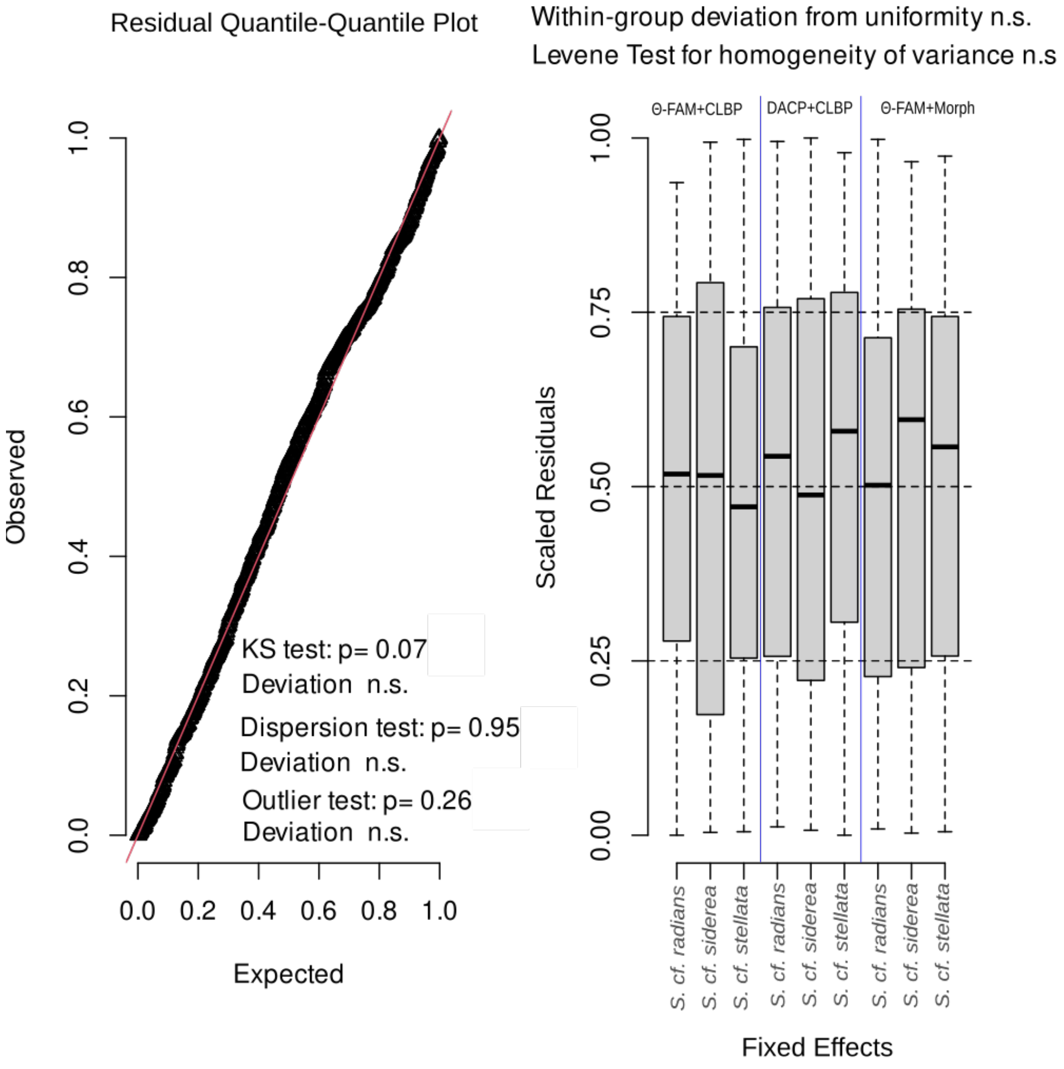

Fig. S11. Residual analysis of GLM comparing the performance of classifiers applied to different descriptors sampled from specimens in *Siderastrea* spp. using the R package DHARMa(42). a) Quantile-quantile (QQ) plots of observed and expected residuals distributions, computed from 1000 simulations. b) Uniformity and variance homogeneity test of re-scaled residuals within and among predictors. See the Results section in the Main Text for further information on the statistical analysis and the caption of Fig. S10 for more details on the residual analysis. CLBP = Complete Linear Binary Patterns, Morph = morphometric descriptors, DAPC = (linear) Discriminant Analysis of Principal Components.

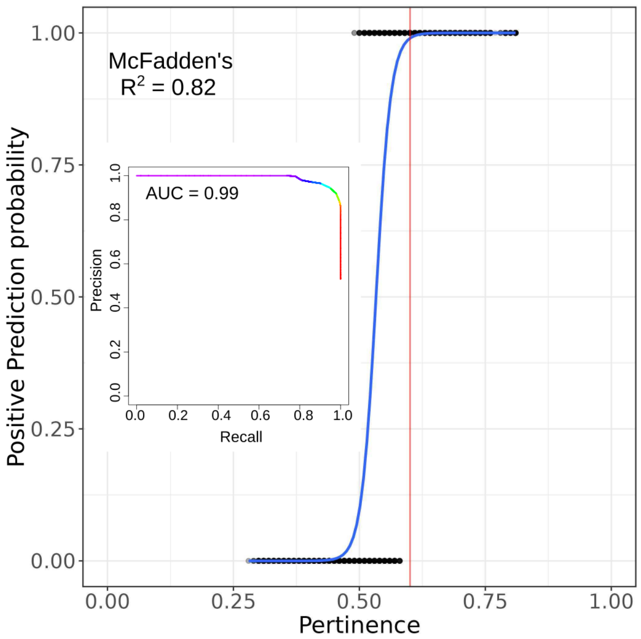

(a)

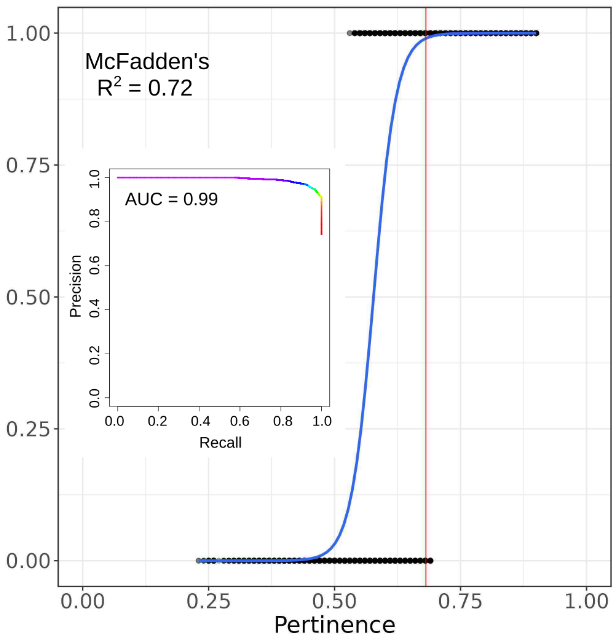

(b)

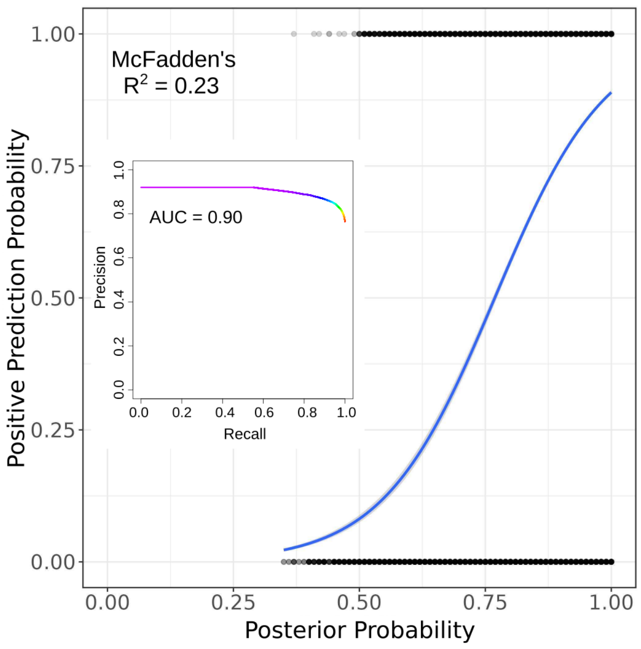

(c)

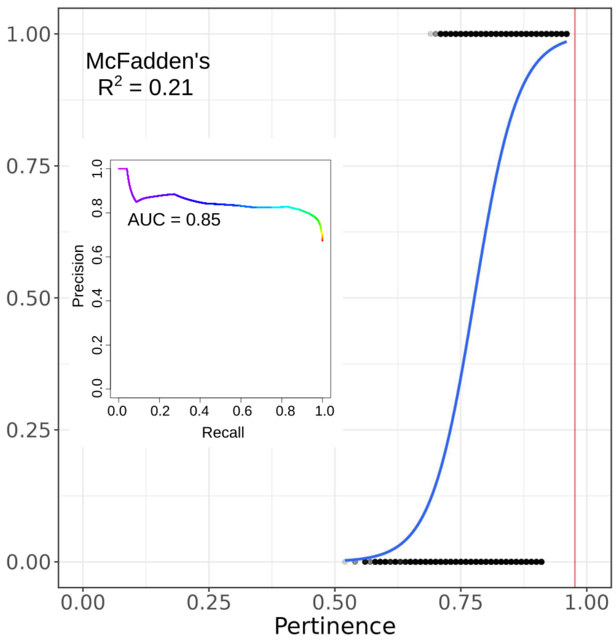

(d)

Fig. S12. Logistic regressions adjusted to the binarized outcomes (points) of all replicates of the 5-fold cross-validation experiments computed from: (a) pertinence scores obtained from Complete Linear Binary Patterns (CLBP) sampled from *Porites* spp. images and classified using Θ-FAM. Raw data in Fig. S3; (b) pertinence scores - CLBP *Siderastrea* spp. + Θ-FAM. Raw data in Fig. S4; (c) probability scores obtained from CLBP sampled from *Siderastrea* spp. images and classified using (linear) Discriminant Analysis of Principal Components (DAPC). Raw data in Fig. S5; (d) pertinence scores - traditional morphometric descriptors of *Siderastrea* spp. + Θ-FAM. Raw data in Fig. S6. Red lines correspond to the theoretical score yielding a classification success of 99%, estimated using the parameters of the corresponding logistic regression. This line is absent in (c) because the estimate was greater than 1. Fit was highly significant in every case, but the regression's predictive value varied with genus, image treatment and classification strategy as indicated by McFadden's pseudo-R^2 included in each subfigure. Insets show the corresponding Precision-Recall curve. Note that in (c) maximum precision is not achieved even at the highest classification score. AUC = area under the curve.

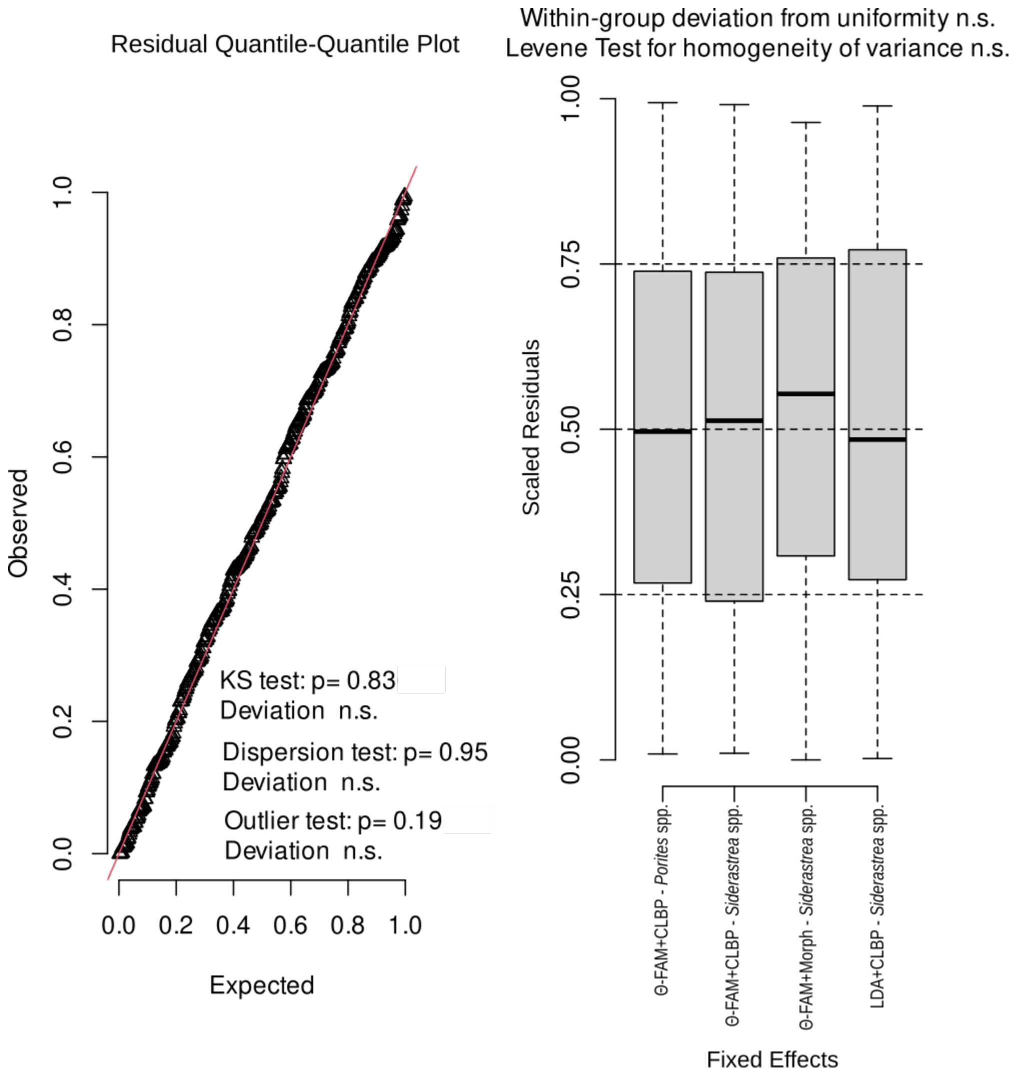

Fig. S13. Residual analysis of GLM comparing the performance of applied classifiers using the R package DHARMa(42). Areas under the Precision-Recall curves computed from binary logistic regressions trained on data from each replicate of the 5-fold cross-validation experiment were used as response variable. a) Quantile-quantile (QQ) plot of observed and expected residuals distributions, computed from 1000 simulations. b) Uniformity and variance homogeneity test of re-scaled residuals within and among predictors. See the Results section in the Main Text for further information on the statistical analysis and the caption of Fig. S10 for more details on the residual analysis. CLBP = Complete Linear Binary Patterns, Morph = morphometric descriptors, DAPC = (linear) Discriminant Analysis of Principal Components.

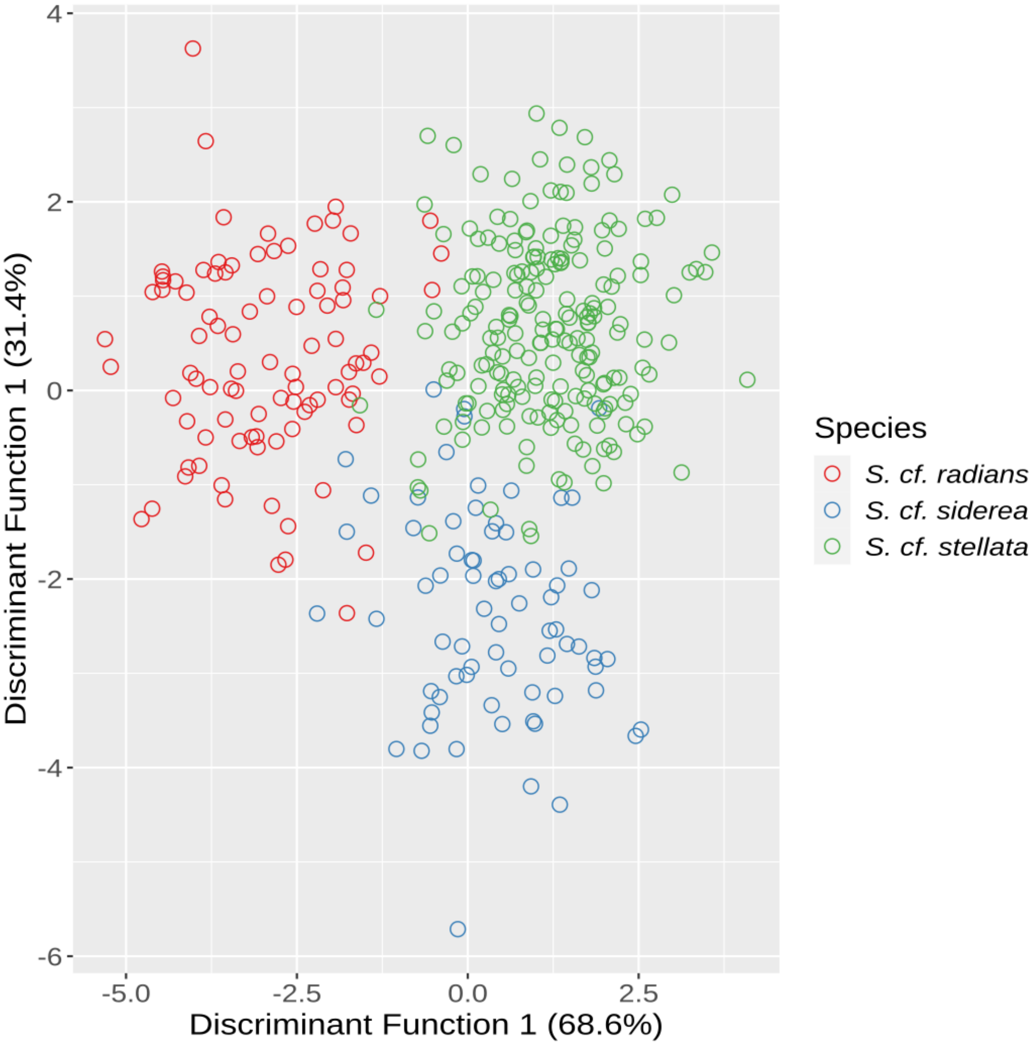

Fig. S14. Morphospace defined by the two discriminant functions generated by the supervised Linear Discriminant Analysis of 100 Principal Components (DPCA), retained by the cross-validation procedure implemented in *adegenet*, and applied to CLBP sampled from *Siderastrea* spp. images.

Table S1. Summary of morphometric characters. See S5 for boxplots.

Level	Type	Character
Corallite	Continuous (mm)	1. Corallite diameter
		2. Collumela diameter
		3. Mean thickness of first cycle septa
		4. Mean length of first cycle septa
	Count	5. Number of septa
	Binary (1/0)	6. 4th septum present/absent
		7. 4th septum complete/incomplete
		8. 5th septum present/absent
		9. 5th septum complete/incomplete
		10. 6th septum present/absent
		11. 6th septum complete/incomplete
Colony	Continuous (mm)	12. Min. distance between collumelae
		13. Max. distance between collumelae
	Binary (1/0)	14. Presence/Absence of intratentacular budding
		15. Presence/Absence of septal continuity

Table S2. Performance comparison of three different combinations of image quantification and classification strategies applied to *Siderastrea* spp. using Generalized Linear Mixed Model (GLMM) fit via maximum-likelihood to the results of the 5-fold cross-validation experiments. Each combination was considered a level in the fixed effect factor *Image Treatment* and each species as a level of the fixed factor *Species*. Bayesian Information Criterion preferred a model with an interaction term between the two fixed factors. All effects were highly significant. CLBP = Complete Linear Binary Patterns, Morph = morphometric descriptors, DAPC = (linear) Discriminant Analysis of Principal Components.

Predictors	Estimates	C.I.	*p*-value
Intercept	0.62	0.61 – 0.63	$<$**0.001**
Morph+θ-FAM	-0.09	-0.10 – -0.08	$<$**0.001**
CLBP+DAPC	-0.03	-0.04 – -0.02	$<$**0.001**
S. cf. *siderea*	0.06	0.05 – 0.08	$<$**0.001**
S. cf. *stellata*	0.06	0.04 – 0.07	$<$**0.001**
Morph+θ-FAM x *S.* cf. *siderea*	-0.22	-0.24 – -0.20	$<$**0.001**
CLBP+DAPC x *S.* cf. *siderea*	-0.18	-0.20 – -0.16	$<$**0.001**
Morph+θ-FAM x *S.* cf. *stellata*	0.03	0.01 – 0.05	**0.002**
CLBP+DAPC x *S.* cf. *stellata*	0.05	0.03 – 0.07	$<$**0.001**
Observations	900		

Table S3. Pairwise comparisons among interactions of fixed factors (*Image Tretatment* x *Species*) levels using Tukey's Honest Significant Difference (HSD) test applied to least-squares means estimated via maximum-likelihood Generalized Linear Mixed Model (GLMM). True Skill Statistic (TSS), computed for each replicate of the 5-fold cross validation experiment, was used as response variables. *P-values* corresponding to significant contrasts are marked in boldface. See main text for further details. CLBP = Complete Linear Binary Patterns, Morph = morphometric descriptors, DAPC = (linear) Discriminant Analysis of Principal Components.

Contrast	Estimate	S.E.	D.F.	*t* ratio	*p*-value
CLBP+Θ-FAM *S.* cf. *radians* x Morph+Θ-FAM. *S.* cf. *radians*	0.0890	0.0062	886	14.255	<**0.001**
CLBP+Θ-FAM *S.* cf. *radians* x CLBP+DAPC *S.* cf. *radians*	0.0292	0.0070	886	4.175	**0.0011**
CLBP+Θ-FAM *S.* cf. *radians* x CLBP+Θ-FAM *S.* cf. *siderea*	-0.0649	0.0057	886	-11.374	<**0.001**
CLBP+Θ-FAM *S.* cf. *radians* x Morph+Θ-FAM. *S.* cf. *siderea*	0.2439	0.0058	886	41.743	<**0.001**
CLBP+Θ-FAM *S.* cf. *radians* x CLBP+DAPC *S.* cf. *siderea*	0.1438	0.0065	886	22.274	<**0.001**
CLBP+Θ-FAM *S.* cf. *radians* x CLBP+Θ-FAM *S.* cf. *stellata*	-0.0556	0.0063	886	-8.780	<**0.001**
CLBP+Θ-FAM *S.* cf. *radians* x Morph+Θ-FAM. *S.* cf. *stellata*	0.0049	0.0065	886	0.752	0.9980
CLBP+Θ-FAM *S.* cf. *radians* x CLBP+DAPC *S.* cf. *stellata*	-0.0800	0.0074	886	-10.875	<**0.001**
Morph+Θ-FAM. *S.* cf. *radians* x CLBP+DAPC *S.* cf. *radians*	-0.0598	0.0071	886	-8.385	<**0.001**
Morph+Θ-FAM. *S.* cf. *radians* x CLBP+Θ-FAM *S.* cf. *siderea*	-0.1539	0.0059	886	-26.160	<**0.001**
Morph+Θ-FAM. *S.* cf. *radians* x Morph+Θ-FAM. *S.* cf. *siderea*	0.1549	0.0060	886	25.759	<**0.001**
Morph+Θ-FAM. *S.* cf. *radians* x CLBP+DAPC *S.* cf. *siderea*	0.0548	0.0066	886	8.293	<**0.001**
Morph+Θ-FAM. *S.* cf. *radians* x CLBP+Θ-FAM *S.* cf. *stellata*	-0.1446	0.0065	886	-22.270	<**0.001**
Morph+Θ-FAM. *S.* cf. *radians* x Morph+Θ-FAM. *S.* cf. *stellata*	-0.0841	0.0067	886	-12.598	<**0.001**
Morph+Θ-FAM. *S.* cf. *radians* x CLBP+DAPC *S.* cf. *stellata*	-0.1690	0.0075	886	-22.553	<**0.001**
CLBP+DAPC *S.* cf. *radians* x CLBP+Θ-FAM *S.* cf. *siderea*	-0.0941	0.0067	886	-14.108	<**0.001**
CLBP+DAPC *S.* cf. *radians* x Morph+Θ-FAM. *S.* cf. *siderea*	0.2147	0.0068	886	31.643	<**0.001**
CLBP+DAPC *S.* cf. *radians* x CLBP+DAPC *S.* cf. *siderea*	0.1146	0.0073	886	15.659	<**0.001**
CLBP+DAPC *S.* cf. *radians* x CLBP+Θ-FAM *S.* cf. *stellata*	-0.0848	0.0072	886	-11.755	<**0.001**
CLBP+DAPC *S.* cf. *radians* x Morph+Θ-FAM. *S.* cf. *stellata*	-0.0243	0.0074	886	-3.290	**0.0287**
CLBP+DAPC *S.* cf. *radians* x CLBP+DAPC *S.* cf. *stellata*	-0.1092	0.0081	886	-13.436	<**0.001**
CLBP+Θ-FAM *S.* cf. *siderea* x Morph+Θ-FAM. *S.* cf. *siderea*	0.3088	0.0055	886	56.586	<**0.001**
CLBP+Θ-FAM *S.* cf. *siderea* x CLBP+DAPC *S.* cf. *siderea*	0.2087	0.0061	886	34.163	<**0.001**
CLBP+Θ-FAM *S.* cf. *siderea* x CLBP+Θ-FAM *S.* cf. *stellata*	0.0093	0.0060	886	1.556	0.8284
CLBP+Θ-FAM *S.* cf. *siderea* x Morph+Θ-FAM. *S.* cf. *stellata*	0.0698	0.0062	886	11.304	<**0.001**
CLBP+Θ-FAM *S.* cf. *siderea* x CLBP+DAPC *S.* cf. *stellata*	-0.0151	0.0071	886	-2.135	0.4501
Morph+Θ-FAM. *S.* cf. *siderea* x CLBP+DAPC *S.* cf. *siderea*	-0.1001	0.0062	886	-16.049	<**0.001**
Morph+Θ-FAM. *S.* cf. *siderea* x CLBP+Θ-FAM *S.* cf. *stellata*	-0.2995	0.0061	886	-49.021	<**0.001**
Morph+Θ-FAM. *S.* cf. *siderea* x Morph+Θ-FAM. *S.* cf. *stellata*	-0.2390	0.0063	886	-37.924	<**0.001**
Morph+Θ-FAM. *S.* cf. *siderea* x CLBP+DAPC *S.* cf. *stellata*	-0.3239	0.0072	886	-45.220	<**0.001**
CLBP+DAPC *S.* cf. *siderea* x CLBP+Θ-FAM *S.* cf. *stellata*	-0.1994	0.0067	886	-29.769	<**0.001**
CLBP+DAPC *S.* cf. *siderea* x Morph+Θ-FAM. *S.* cf. *stellata*	-0.1389	0.0069	886	-20.206	<**0.001**
CLBP+DAPC *S.* cf. *siderea* x CLBP+DAPC *S.* cf. *stellata*	-0.2238	0.0077	886	-29.174	<**0.001**
CLBP+Θ-FAM *S.* cf. *stellata* x Morph+Θ-FAM *S.* cf. *stellata*	0.0605	0.0068	886	8.952	<**0.001**
CLBP+Θ-FAM *S.* cf. *stellata* x CLBP+DAPC *S.* cf. *stellata*	-0.0244	0.0076	886	-3.219	**0.0360**
Morph+Θ-FAM. *S.* cf. *stellata* x CLBP+DAPC *S.* cf. *stellata*	-0.0849	0.0077	886	-10.989	<**0.001**

Table S4. Performance comparison among four image treatment strategies employed in this study using Generalized Linear Model (GLM) fit via maximum-likelihood. Areas under the Precision-Recall curves computed from binary logistic regressions trained on data from each replicate of the 5-fold cross-validation experiment were used as response variable. All effects were highly significant. See main text for further details. CLBP = Complete Linear Binary Patterns, Morph = morphometric descriptors, DAPC = (linear) Discriminant Analysis of Principal Components.

Predictors	Estimates	CI	p
Intercept	0.99	0.99 – 0.99	$<$**0.001**
CLBP+Θ-FAM - *Siderastrea* spp.	0.00	0.00 – 0.00	$<$**0.001**
Morph+Θ-FAM - *Siderastrea* spp.	-0.14	-0.15 – -0.14	$<$**0.001**
CLBP+DAPC - *Siderastrea* spp.	-0.10	-0.11 – -0.10	$<$**0.001**
Observations	400		

Table S5. Pairwise comparisons among levels of fixed-effect (*Image Tretament*) least-squares means estimated via maximum-likelihood Generalized Linear Model (GLM) using Tukey's Honest Significant Difference (HSD) test. Areas under the precision-recall curves computed for each replicate of the re-shuffled 5-fold cross validation experiment after binarization were used as response variables. All contrasts were highly significant. See main text for further details. CLBP = Complete Linear Binary Patterns, Morph = morphometric descriptors, DAPC = (linear) Discriminant Analysis of Principal Components.

Contrast	Estimate	S.E.	D.F.	*t* ratio	*p*-value
CLBP+Θ-FAM - *Porites* spp. x CLBP+Θ-FAM - *Siderastrea* spp.	-0.0027	0.0005	392	-5.388	$<$**0.001**
CLBP+Θ-FAM - *Porites* spp. x Morph+Θ-FAM. - *Siderastrea* spp.	0.1443	0.0018	392	79.498	$<$**0.001**
CLBP+Θ-FAM - *Porites* spp. x CLBP+DAPC - *Siderastrea* spp.	0.1032	0.0019	392	53.290	$<$**0.001**
CLBP+Θ-FAM - *Siderastrea* spp. x Morph+Θ-FAM. - *Siderastrea* spp.	0.1471	0.0018	392	82.275	$<$**0.001**
CLBP+Θ-FAM - *Siderastrea* spp. x CLBP+DAPC - *Siderastrea* spp.	0.1059	0.0019	392	55.462	$<$**0.001**
Morph+Θ-FAM - *Siderastrea* spp. x CLBP+DAPC - *Siderastrea* spp.	-0.0412	0.0026	392	-15.942	$<$**0.001**

Table S6. Brief review of papers employing morphometrics to draw boundaries both within and among coral species. Technique: ANOVA = Analysis of Variance, CA = Cluster Analysis, CCA = Canonical Correlation Analysis, CDA = Canonical Discriminant Function Analysis, CVA = Canonical Variate Analysis, LDA = Linear Discriminant Analysis, MANOVA = Multivariate ANOVA, MW = Mann-Whitney test, PERMANOVA = Permutational Multivariate Analysis of Variance, SMDCA = Stepwise Multivariate Canonical Discriminant Analysis, SPCA = Sheared Principal Component Analysis. Use: E = Exploratory, C = Classifier (cross-validation was employed to measure classification success). PC = Pseudoreplication Control.

Authorship	Year	Taxon	Technique	Use	PC	Notes
Budd, 1990(43)	1990	Several species	CDA, LDA, SPCA	E	Yes	Accounted for classification success, but no cross-validation procedure was employed
Weil, 1992(44)	1992	*Porites* spp.	SMDCA	E	No	Accounted for classification success, but no cross-validation procedure was employed
Amaral, 1994(45)	1994	*Montastrea cavernosa*	CVA	E	No	
Miller, 1994(46)	1994	*Platygyra* spp.	CDA	E	No	
Weil & Knowlton, 1994(47)	1994	*Montastrea annularis* complex	SMDCA	E	No	Synonimized to *Orbicella* spp.
Zilberberg & Edmunds, 1999(48)	1999	*Montastraea franksi*	PCA	E	No	
Manica and Carter, 2000(49)	2000	*Montastraea annularis*	ANOVA, LDA, MANOVA, PCA	C	No	Synonimized to *Orbicella annularis*. Authors did not report cross-validation success rates; although they claim that LDA was employed, axis in scatterplots were labeled as Canonical Variates
Todd et al., 2001(50)	2001	*Favia speciosa*	CDA, MANOVA, PCA	E	Yes	Control for pseudoreplication but only in univariate analysis
Carlon & Budd, 2002(51)	2002	*Favia fragum*	CDA, LDA	E	No	CDA cross-validation was employed to establish cutoff points along the dendrogram in order to delimit morphotypes
Pandolfi et al., 2002(52)	2002	*Montastraea annularis* complex	CA, CDA	E	No	Synonimized to *Orbicella* spp.
Wolstenholme et al. 2003(53)	2003	*Acropora humilis*	CA, PCA	E	No	
Budd & Pandolfi, 2004(54)	2004	*Montastrea annularis*	CDA, Tukey HSD test	E	No	Synonimized to *Orbicella annularis*
Todd et al., 2004(55)	2004	*Favia speciosa*, *Diploastrea heliopora*	ANOVA, PCA	E	No	
Mangubhai et al., 2007(56)	2007	*Platygyra daedalea*	ANOVA, CDA	C	No	
Forsman et al., 2010(57)	2010	*Montipora* spp.	CDA, MANOVA, PCA	E	No	
Budd et al., 2012(58)	2012	*Montastraea cavernosa*	CA, CDA	E	No	CDA cross-validation was employed to establish cutoff points along the dendrogram in order to delimit morphotypes
López-Pérez, 2013(59)	2013	*Porites* spp.	ANOVA, CDA, PERMANOVA	E	Yes	
Menezes, 2013(19)	2013	*Siderastrea* spp.	CDA, MW	E	No	
Menezes, 2014(60)	2014	*Siderastrea* spp.	CDA, PERMANOVA	E	No	
Zhang, 2016(61)	2016	*Plasmoporella* spp.	CA, CCA, LDA, PCA	E	No	Accounted for classification success of LDA, but no cross-validation procedure was employed
García et al., 2017(21)	2017	*Siderastrea* spp.	ANOVA, CDA	E	No	
Grinyó et al., 2018(62)	2018	*Paramuricea macrospina*	PCA, PERMANOVA	E	No	
Tisthammer & Richmond, 2018(63)	2018	*Porites lobata*	CDA, PCA, PERMANOVA	C	No	

Table S7. List of vouchers used in this study

Locality	Site	Depth (m)	Lat.	Long.	Species	Voucher
Búzios	Praia da Tartaruga	-22.80	-41.91	0.5-2.0	*Siderastrea* cf. *stellata*	217
						218
						219
						220
						221
						222
						224
						225
						226
						227
						228
						229
						230
						231
						232
						233
						234
Aracruz	Praia do Pichado	-20.01	-40.16	0.5-1.5	*Siderastrea* cf. *radians*	241
						252
					Siderastrea cf. *stellata*	238
						239
						240
						242
						243
						244
						245
						246
						247
						248
						249
						250
						251
Abrolhos	Caldeiras	-18.20	-38.87	4.0-5.5	*Porites astreoides*	322
					Siderastrea cf. *radians*	324
						328
						329
					Siderastrea cf. *stellata*	321
						327
						333
	Chapeirinhos da Sueste	-18.20	-38.79	8.0-12.0	*Siderastrea* cf. *siderea*	291
						298
						301
					Siderastrea cf. *stellata*	293
						295
						300
						305
						306
						308
	Chapeirões do Sul	-18.07	-38.73	21.0-23.0	*Siderastrea* cf. *siderea*	277
						280
						281
						285
					Siderastrea cf. *stellata*	284

Continued on next page

Table S7 – continued from previous page

Locality	Site	Depth (m)	Lat.	Long.	Species	Voucher
	Redonda	-18.00	-38.89	5.0-6.0	*Porites astreoides*	275
					Porites branneri	260
						267
						270
					Siderastrea cf. *stellata*	262
						263
	Chapeirão	-17.90	-38.83	14.0-15.0	*Porites astreoides*	318
					Siderastrea cf. *stellata*	314
						315
						316
Boipeba	Ponta dos Castelhanos	-13.67	-38.89	0.5-2.0	*Porites astreoides*	020
					Siderastrea cf. *siderea*	031
					Siderastrea cf. *stellata*	015
						017
						022
						025
	Tassimirim	-13.58	-38.91	0.5-4.0	*Porites astreoides*	074
						076
					Porites branneri	033
						037
						045
						056
						061
						063
						064
						068
						071
					Siderastrea cf. *radians*	041
						044
						077
					Siderastrea cf. *stellata*	038
						048
						051
						065
						067
						069
						072
						079
	Praia de Moreré	-13.58	-38.91	0.5-2.0	*Porites astreoides*	011
Tamandaré	Igreja de São Pedro	-8.74	-35.09	0.5-2.0	*Porites astreoides*	123
						125
	Praia de Campas	-8.74	-35.08	0.5-2.0		115
						118
					Siderastrea cf. *radians*	089
						091
						096
						105
						112
						116
Maxaranguape	Parrachos de Maracajau	-5.39	-35.25	0.5-2.0	*Porites astreoides*	198
						201

Continued on next page

Table S7 – continued from previous page

Locality	Site	Depth (m)	Lat.	Long.	Species	Voucher
					Siderastrea cf. *siderea*	181
						185
					Siderastrea cf. *stellata*	164
						170
						179
						183
						184
						190
					Porites astreoides	133
						137
					Siderastrea cf. *radians*	138
						147
						150
					Siderastrea cf. *siderea*	131
						153
					Siderastrea cf. *stellata*	128
						129
						148
						151

References

1. Pallas PS (1766) *Elenchus zoophytorum sistens generum adumbrationes generaliores et specierum cognitarum succintas descriptiones, cum selectis auctorum synonymis.* (Apud Petrum van Cleef, Hagae-Comitum), p. 498.
2. Milne-Edwards H (1854) *A monograph of the British fossil corals.* (Palaeontographical society) Vol. 1.
3. Milne-Edwards H (1857) *Histoire naturelle des coralliaires ou polypes proprement dits.* (Librairie encyclopédique de Roret) Vol. 2.
4. de Blainville HMD (1830) Zoophytes. *Dictionnaire des Sciences naturelles, dans lequel on traite méthodiquement des différents êetres de la nature* 60:1–546.
5. Duerden JE (1902) *West Indian madreporarian polyps.* (US Government Printing Office) Vol. 8.
6. Verrill AE (1868) *Notice of the corals and echinoderms collected by Prof. CF Hartt, at the Abrolhos Reefs, province of Bahia, Brazil, 1867.*
7. Gregory JW (1895) Contributions to the palaeontology and physical geology of the West Indies. *Quarterly Journal of the Geological Society* 51(1-4):255–NP.
8. Vaughan TW (1919) *Fossil corals from Central America, Cuba, and Porto Rico: with an account of the American Tertiary, Pleistocene, and recent coral reefs.* (US Government Printing Office) Vol. 103.
9. Budd AF, Guzman HM (1994) *Siderastrea glynni*, a new species of scleractinian coral (Cnidaria:Anthozoa) from the Eastern Pacific. *Proceedings of the Biological Society of Washington* 107(4):591.
10. Reyes J, Santodomingo N, Flórez P (2010) *Corales escleractinios de Colombia.* (Instituto de Investigaciones Marinas y Costeras" José Benito Vives de Andréis,") No. 943.
11. Vaughan TW, Wells JW (1943) Revision of the suborders families, and genera of the Scleractinia in *Revision of the Suborders Families, and Genera of the Scleractinia*, eds. Vaughan TW, Wells JW. (Geological Society of America) Vol. 44.
12. Forsman Z, Guzman HM, Chen CA, Fox G, Wellington GM (2005) An ITS region phylogeny of *Siderastrea* (Cnidaria:Anthozoa): is *S. glynni* endangered or introduced? *Coral Reefs* 24(2):343–347.
13. LaJeunesse TC, Forsman ZH, Wham DC (2016) An Indo-West Pacific 'zooxanthella' invasive to the western Atlantic finds its way to the Eastern Pacific via an introduced Caribbean coral. *Coral Reefs* 35(2):577–582.
14. Glynn PW, Grassian B, Kleemann KH, Maté JL (2016) The true identity of *Siderastrea glynni* Budd & Guzmán, 1994, a highly endangered eastern Pacific scleractinian coral. *Coral Reefs* 35(4):1399–1404.
15. Laborel J (1969) Madreporaires et hydrocoralliares recifaux des cotes bresiliennes. systematique, ecologie. repartition verticale et geographique. *Results Scientifique du Campagne de Calypso* 9(25):171–229.
16. Veron JEN (1995) *Corals in space and time: the biogeography and evolution of the Scleractinia.* (Cornell University Press).
17. Maida M, Ferreira BP (1997) Coral reefs of Brazil: an overview in *Proceedings of the 8th International Coral Reef Symposium.* (Smithsonian Tropical Research Institute Panamá), No. 263, p. 74.

18. Castro CB, Pires DO (2001) Brazilian coral reefs: what we already know and what is still missing. *Bulletin of Marine Science* 69(2):357–371.
19. Menezes NM, Neves EG, Barros F, Kikuchi RKP, Johnsson R (2013) Intracolonial variation in *Siderastrea* de Blainville, 1830 (Anthozoa, Scleractinia): taxonomy under challenging morphological constraints. *Biota Neotropica* 13(1):108–116.
20. Echeverría C, Pires D, Medeiros M, Castro C (1997) Cnidarians of the Atol das Rocas, Brazil in *Proceedings of the 8th International Coral Reef Symposium.* Vol. 2, pp. 443–446.
21. García NAC, et al. (2017) Comparative molecular and morphological variation analysis of *Siderastrea* (Anthozoa, Scleractinia) reveals the presence of *Siderastrea stellata* in the Gulf of Mexico. *The Biological Bulletin* 232(1):58–70.
22. Neves E, Silveira F, Johnsson R (2016) Cnidaria, Scleractinia, Siderastreidae, *Siderastrea siderea* (Ellis and Solander, 1786): Hartt Expedition and the first record of a Caribbean siderastreid in tropical Southwestern Atlantic. *Check List* 6:505.
23. Santos M, Amaral F, Hernández M, Knowlton N, Jara J (2004) Variação morfológica de *Favia gravida* Verrill, 1868 e *Siderastrea stellata* Verrill, 1868 (Cnidaria, Scleractinia): aspectos esqueléticos. *Boletim do Museu Nacional de Zoologia* 517:1–9.
24. Werner T (1996) Ph.D. thesis (University of Maryland).
25. Neves EG, Da Silveira FL (2003) Release of planula larvae, settlement and development of *Siderastrea stellata* Verrill, 1868 (Anthozoa, Scleractinia). *Hydrobiologia* 501(1-3):139–147.
26. Neves EG, Andrade SCS, da Silveira FL, Solferini VN (2008) Genetic variation and population structuring in two brooding coral species (*Siderastrea stellata* and *Siderastrea radians*) from Brazil. *Genetica* 132(3):243–254.
27. Nunes F, Norris R, Knowlton N (2009) Implications of isolation and low genetic diversity in peripheral populations of an amphi-Atlantic coral. *Molecular Ecology* 18(20):4283–4297.
28. Szmant AM (1986) Reproductive ecology of Caribbean reef corals. *Coral Reefs* 5(1):43–53.
29. Harrison PL (1990) Reproduction, dispersal and recruitment of scleractian corals. *Coral Reefs.*
30. Barros MML, Oliveira Pires D (2003) Sexual reproduction of the brazilian reef coral *Siderastrea stellata* Verrill 1868 (Anthozoa, Scleractinia). *Bulletin of Marine science* 73(3):713–724.
31. Barros MML, Pires DO (2006) Colony size-frequency distributions among different populations of the scleractinan coral *Siderastrea stellata* in Southwestern Atlantic: implications for life history patterns. *Brazilian Journal of Oceanography* 54(4):213–223.
32. Kriegeskorte N, Golan T (2019) Neural network models and deep learning. *Current Biology* 29(7):R231–R236.
33. Zadeh LA (1965) Fuzzy sets. *Information and Control* 8(3):338–353.
34. Graña M (2009) Lattice computing and natural computing. *Neurocomputing* 10-12(72):2065–2066.
35. Buckley JJ, Hayashi Y (1993) Numerical relationships between neural networks, continuous functions, and fuzzy systems. *Fuzzy Sets and Systems* 60(1):1–8.
36. Zeng W, Li H (2006) Inclusion measures, similarity measures, and the fuzziness of fuzzy sets and their relations. *International Journal of Intelligent Systems* 21(6):639–653.
37. Bustince H, Mohedano V, Barrenechea E, Pagola M (2006) A method for constructing V. Young's fuzzy subsethood measures and fuzzy entropies in *2006 3rd International IEEE Conference Intelligent Systems.* (IEEE, London, UK), pp. 208–214.
38. Esmi E, Sussner P, Valle ME, Sakuray F, Barros L (2012) Fuzzy associative memories based on subsethood and similarity measures with applications to speaker identification in *Hybrid Artificial Intelligent Systems*, eds. Corchado E, et al. (Springer Berlin Heidelberg, Berlin, Heidelberg), pp. 479–490.
39. Sussner P, Esmi EL, Villaverde I, Graña M (2012) The Kosko subsethood fuzzy associative memory (KS-FAM): Mathematical background and applications in computer vision. *Journal of Mathematical Imaging and Vision* 42(2):134–149.
40. Esmi E, Sussner P, Sandri S (2014) An introduction to tunable equivalence fuzzy associative memories in *2014 IEEE International Conference on Fuzzy Systems (FUZZ-IEEE).* pp. 1604–1611.
41. Esmi E, Sussner P, Bustince Sola H, Fernandez J (2015) Theta-fuzzy associative memories (Theta-FAMs). *IEEE Transactions on Fuzzy Systems* 23(2):313–326(14).
42. Hartig F (2021) *DHARMa: Residual Diagnostics for Hierarchical (Multi-Level / Mixed) Regression Models.* R package version 0.4.4.
43. Budd AF (1990) Longterm patterns of morphological variation within and among species of reef corals and their relationship to sexual reproduction. *Systematic Botany* 15(1):150–165.
44. Weil E (1992) Genetic and morphological variation in Caribbean and Eastern Pacific *Porites* (Anthozoa, Scleractinia). Preliminary results in *Proceedings of the 7th International Coral Reef Symposium.* Vol. 2, pp. 643–656.
45. Amaral F (1994) Morphological variation in the reef coral *Montastrea cavernosa* in Brazil. *Coral Reefs* 13(113-117).
46. Miller K (1994) Morphological variation in the coral genus *Platygyra*: Environmental influences and taxonomic implications. *Marine Ecology Progress Series* 110:19–28.
47. Weil, Knowlton (1994) A multi-character analysis of the Caribbean coral *Montastraea annularis* (Ellis and Solander, 1786) and its two sibling species, *M. faveolata* (Ellis and Solander, 1786) and *M. franksi* (Gregory, 1895). *Bulletin of Marine Science* 55(1):175. TY - JOUR RP - Not in File.
48. Zilberberg C, Edmunds PJ (1999) Patterns of skeletal structure variability in clones of the reef coral *Montastraea franksi.* *Bulletin of Marine Science* 64(2):373–381.

49. Manica A, Carter RW (2000) Morphological and fluorescence analysis of the *Montastraea annularis* species complex in Florida. *Marine Biology* 137(5):899–906.
50. Todd PA, Sanderson PG, Chou LM (2001) Morphological variation in the polyps of the scleractinian coral *Favia speciosa* (Dana) around Singapore. *Hydrobiologia* 444(1):227–235. 10.1023/A:1017570100029.
51. Carlon DB, Budd Ann F (2002) Incipient specitaion across a depth gradient in a scleractinian coral. *Evolution* 56(11):2227–2242.
52. Pandolfi JM, Lovelock CE, Budd AF (2002) Character release following extinction in a Caribbean reef species complex. *Evolution* 56(3):479–501.
53. Wolstenholme J, Wallace CC, Chen CA (2003) Species boundaries within the *Acropora humilis* species group (Cnidaria; Scleractinia): a morphological and molecular interpretation of evolution. *Coral Reefs* 22:155–166. Lido.
54. Budd AF, Pandolfi JM (2004) Overlapping species boundaries and hybridization within the *Montastraea "annularis"* reef coral complex in the Pleistocene of the Bahama Islands. *Paleobiology* 30(3):396–425.
55. Todd PA, Ladle RJ, Lewin-Koh NJI, Chou LM (2004) Genotype × environment interactions in transplanted clones of the massive corals *Favia speciosa* and *Diploastrea heliopora*. *Marine Ecology Progress Series* 271:167–182. 10.3354/meps271167.
56. Mangubhai S, Souter P, Grahn M (2007) Phenotypic variation in the coral *Platygyra daedalea* in Kenya: morphometry and genetics. *Marine Ecology Progress Series* 345:105–115.
57. Forsman ZH, et al. (2010) Ecomorph or endangered coral? DNA and microstructure reveal Hawaiian species complexes: *Montipora dilatata/flabellata/turgescens* & *M. patula/verrilli*. *PLoS ONE* 5(12):e15021.
58. Budd AF, Nunes FLD, Weil E, Pandolfi JM (2012) Polymorphism in a common Atlantic reef coral (*Montastraea cavernosa*) and its long-term evolutionary implications. *Evolutionary Ecology* 26(2):265–290.
59. López-Pérez RA (2013) Species composition and morphologic variation of *Porites* in the Gulf of California. *Coral Reefs* 32(3):867–878.
60. Menezes N (2014) Morphological variation in the Atlantic genus *Siderastrea* (Anthozoa, Scleractinia). *Papéis Avulsos de Zoologia* 54(16).
61. Zhang F (2016) Recognizing morphospecies in the heliolitid coral *Plasmoporella*. *Palaeoworld* 25(1):32–42.
62. Grinyó J, et al. (2018) Morphological features of the gorgonian *Paramuricea macrospina* on the continental shelf and shelf edge (Menorca Channel, Western Mediterranean Sea). *Marine Biology Research* 14(1):30–40.
63. Tisthammer KH, Richmond RH (2018) Corallite skeletal morphological variation in Hawaiian *Porites lobata*. *Coral Reefs* 37(2):445–456.
