# Supplementary material for: AI-based coral species discrimination: A case study of the Siderastrea Atlantic Complex
Source: PLoS One. 2024 Dec 11;19(12):e0312494. doi: 10.1371/journal.pone.0312494 (PMC11634003; doi:10.1371/journal.pone.0312494)
Supplement: Attachment — Submitted filename: Response to Reviewers.pdf [file pone.0312494.s002.pdf]

13 April 2023

Dear Dr. Zapalski,

As per your re-submission encouragement, please find attached a new version of the paper “Hard coral species: I know them when I see them”, retitled as “AI based coral species discrimination: a case study of the Siderastrea Atlantic Complex”. We have addressed all reviewer’s comments below and incorporated many changes into this new version.

We hope you find this manuscript worthy of publication in PLOS One.

Sincerely,

Dr. Marcos Soares Barbeitos
Associate Professor

Departamento de Zoologia - Sala 360
Setor de Ciências Biológicas
Universidade Federal do Paraná
Caixa Postal 19020
Curitiba, PR 81531-980
Brazil

Phone: (55 41) 3361-1634

Reviewer #1

“The first is that the study lacks a clear hypothesis in what it is testing. I think the benchmarking comparisons are clear, but it’s not clear what we should be expecting the traditional methods to do with respect to species discrimination, especially since the definition of what a morphospecies means biologically is not addressed. The text, perhaps in the introduction, needs to clearly highlight the limits of inference for each of the approaches tested and the specific hypotheses that the comparisons are targeting.”

We respectfully disagree with the reviewer: we do have a clearly stated hypothesis, namely, that taxonomists perceive differences in Siderastrea coralla that they are unable to translate into meaningful quantitative characters thus confusing unskilled end users. This is clearly depicted in Fig. S1. Computer vision offers an alternative and faster way to capture such differences and fuzzy ANNs are needed as classifiers due to the blurry boundaries among species delimited using these characters. We addressed the reviewers comments about the comparisons in the last paragraph of the introduction, making the purpose of each experiment clearer. We believe, however, that the issue of the biological meaning of morphospecies is tangential to this paper. As stated in the first paragraph, our motivation was rather practical, namely, our inability to discriminate among species using available taxonomic keys.

-

“The second, relatedly, is that the benchmark against traditional morphometric and classification analyses is weak since it has been known for some time that conventional morphology and morphometry based on macromorphological features are unreliable for distinguishing phylogenetically distinct species (see e.g . ...”

We agree with the statement. Nevertheless, coral species were originally described using these characters (as briefly reviewed in the Supplementary Material) and new species descriptions will also be based on these same characters to the extent that is possible. More recent taxonomic treatments of Scleractinian species have included micromorphological and microstructural characters (also sampled using SEMs and therefore likely to be quantifiable by texture descriptors such as CLBP), clearly indicating that species description will continue to rely on morphology for the foreseeable future. We added verbiage to the conclusion to illustrate how texture descriptors (TD) could be applied to those characters.

-

"It is thus unclear what the need is for these tools. It would help to illustrate scenarios in which these approaches will be used, other than citing the oft-used excuse that genetic approaches are costly. If indeed these latter approaches are costly, then the real value of such new tools can be realised if they are benchmarked against genetic discrimination approaches, not with traditional morphological/morphometric tools which we already know are problematic."

We have reformulated the Conclusions in this paper to address the reviewer's objections with respect to genetic delimitation. The extensive application of morphometrics to scientific research involving Scleractinians is clearly documented by our short literature review. The combination of TD and fuzzy ANNs was chosen due to the particularities of species discrimination in Siderastrea, but these approaches may be separately employed, as illustrated by Fig. S11. We believe it should be immediately evident to any experienced researcher how TD would be useful in feature extraction from coral SEMs. Fuzzy ANNs also offer an attractive option when it comes to (not only morphological) species discrimination. If a specimen has **low** pertinence to any previously labeled species, this suggests that the particular specimen probably does not bear traits that can be associated with those listed in taxonomic keys. If the network is confused because those traits offer little discrimination among specimens, as it is seemingly the case with traditional characters, pertinence to all species will be **high**. These are distinct scenarios and both are impossible under a probabilistic framework for the probabilities of assignment to the labelled species must necessarily add up to 1. As we have indicated, therefore concurring with the reviewer, the inability of the network to correctly assign some individuals to the labelled species may result from limited training. The corollary of this affitmation, however, is that a larger training set would improve the performance of the network. As it is, we believe our results are strong enough to corroborate the main points of the paper as stated above hence additional training would only strengthen our conclusions. From our point of view, the use for the tools presented in this paper are rather clear. Additionally, we have outlined a framework to evaluate the performance of discrimination strategies that is much more rigorous than any other published treatment we were able to find.

"Line 155: belonging TO 93 Siderastrea spp. colonies"

Fixed.

"Line 156: photographed"

Fixed.

"Line 331: 'BIC once more supported the modelling of overdispersion and residual analyses showed no deviation from the expected under the chosen model'. This statement needs to be clarified."

We fixed the figure number and modified the sentence to clarify that residual analysis was conducted using DHARMa.

"Line 336: 'performance… was' or 'performances… were'."

Fixed.

Reviewer #2

"1. The results do not make sense to me. How can you compare various techniques for identifying species if you don't know the true identify of each colony? This seems a fundamental flaw to me."

We respectfully differ from the reviewer on this issue for two reasons. First, species limits are clear in the case of Porites, so we did apply the combination of CLBP+Θ-FAM to a situation in which we knew the each colony's species. Second, if species boundaries were clearly defined in the taxonomic keys, there would be no need to try new methods as the keys would suffice. Soft decision boundaries are common in many other areas of knowledge and fuzzy logic was created precisely to deal with such situations.

-

“2. The authors confuse identification with taxonomy. I don’t find it either surprising or interesting that you can train a computer to discriminate among different shapes and textures. However, this falls well short of taxonomy which means giving these things the correct name - not just any name. This requires examination of the type materials, revisiting the synonymies, etc etc”

We agree that the paper is about species discrimination and not taxonomy, and we have reformulated the title, introduction and the discussion in order to eliminate any possible source of confusion. However, our short survey of the published literature reveals that morphometrics is frequently employed by coral biologists. Therefore, training a computer as a classifier of morphological features may be neither surprising nor interesting, but it is certainly useful to that community.

-

“3. The sampling design is unrealistic. Why sample over this scale to test your approach (see comment above)? I would have concentrated on lots of colonies of each putative species from a single site and combined this with more lines of evidence to determine “true” species identify, for example, some molecular technique if available.”

It is easy to realize upon the examination of Fig. S1 that there is a strong biogeographic component to trait variation in Siderastrea spp., therefore, reduced geographic sampling would be ill-advised. As stated in the first paragraph, our motivation stems from the very practical need of identifying species using current taxonomic keys. There is no other source of information, beyond taxonomic keys, that could be accessed in order to conduct more rigorous species assignment. Alas, not even types have been designated for each of the species of the Atlantic complex, which highlights the scarcity of taxonomic resources. Hence, there is no guarantee that genetic analysis would recover species in agreement with taxonomic keys, since taxonomy in this genus is so poorly resolved. We have added more information to the paper conclusions explaining how the approach outlined in this paper may actually help researchers that resort to genetic data in order to delimit species in this genus.

-

“4. The statement “I know it when I see it” would not be accepted in the methods of any paper and I don’t think it should be accepted here.”

We have removed this statement from the paper.
