# Supplementary material for: AI-based coral species discrimination: A case study of the Siderastrea Atlantic Complex
Source: PLoS One. 2024 Dec 11;19(12):e0312494. doi: 10.1371/journal.pone.0312494 (PMC11634003; doi:10.1371/journal.pone.0312494)
Supplement: Attachment — Submitted filename: Response to Reviewers.pdf [file pone.0312494.s003.pdf]

Reviewer #1

1. "The authors started the manuscript by raising a relevant discussion about the challenges of species identification. However, in the way that the first paragraph is presented, it seems that taxonomic descriptions are unreliable. I can see the utility of applying new methods to facilitate and improve species delimitation, but the taxonomic work is not the source of the problem. Furthermore, the term "taxonomic impediment" means a lot more than the difficult faced by end users (which it is not listed within the "Real taxonomic impediments" in the cited work of Ebach et al. 2011) and I don't think the term is properly address by the authors (it is at least oversimplified). Citing Ebach et al. (2011) "It is erroneous to think that taxonomy can be replaced by taxonomic tools or technological surrogates. Such issues are merely applications for taxonomic information."

R. We agree with the reviewer. We cited Ebach et al. 2011 in the context of proper training of end users as a major source of taxonomic impediment. We attempted to clarify this by changing the sentence in Introduction from:

"To be fair, this is certainly a feature of many taxonomic keys and the trouble non-specialists face going through them has been termed 'taxonomic impediment to end users'"

to:

"To be fair, this is certainly a feature of many taxonomic keys and the trouble that those **who lack proper taxonomic training** face going through them has been termed 'taxonomic impediment to end users'"

We also added verbiage to the first paragraph of the Conclusion to stress that the approach used in this paper is not intended to replace taxonomic work and removed the last paragraph to eliminate any suggestion that taxonomic impediment will be eliminated by technology and not by properly trained taxonomists.

2. "I understand the concern raised by Reviewer #2 regarding not knowing the true species identity. Maybe if the analysis were also performed in the type species this issue could be better addressed in the manuscript and it would shed some light on the species delimitation within Siderastrea."

R. We agree with both Reviewers. Unfortunately, there are three major issues here. The first is that, among the Atlantic species, only *Siderastrea glynni* (a synonym of *S. siderea*) has a designated (holo) type specimen. Second, the method requires removing a sample from the corallum hence it could be destructive for smaller type specimens. Third, the evidence presented in S1 Fig suggests that there is considerable geographic variation in diagnostic characters, thus a single holotype per species would not adequately capture the range of morphological variation present in species of this genus.

3. "How to deal with geographic differences? The measurements provide in Figure S1 show, in some cases, a strong divergence between samples from Brazil and Gulf of Mexico. Some species have similar morphology due to environmental constrains and are difficult to separate based solely on morphological characters. I wonder how the proposed analysis would handle these issues."

R. That is precisely the role of fuzzy logic. This approach acknowledges that hard boundaries among human designated categories (species, in our case) may actually not exist and incorporates this uncertainty into the discrimination process. If geographic and/or environmentally introduced

variation is such that hard bounds cannot really be drawn, pertinence scores will also reflect that as This was clearly demonstrated in the case of *Porites* (see Fig 2 and S6 Fig): even though there is no doubt about species assignment in this genus due to the obvious differences in color and colony shape, discrimination would be much harder if one was to rely solely on corallite morphology, as evidenced in S2 Fig.

4. “Furthermore, an important question that could be more clearly addressed about the utility of the tools (a concern raised by reviewer #1) is if the method helped to clarify undistinguishable specimens.”

R. We believe to have clearly addressed this concern in the paper by showing that the combination of CLBP and Θ-FAM outperformed the alternative classifier most commonly applied to such problems: the instability of DAPC (an improved variant of LDA), uncovered by rigorous application of ML performance evaluation, shows that the seemingly satisfactory (and certainly overrated) results in S14 Fig are a consequence of inadequate application of the method. We invite the reviewers to appreciate the fact that we could have very well identified specimens to the best of our knowledge and claimed success after those results, as our brief survey suggests that most researchers would do. Instead, we attempted to incorporate the fuzziness in morphological boundaries introduced by geographic/environmental variation, compounded with our very uncertainty about specimen identification into the discrimination process, not as an attempt to devise a foolproof method of species discrimination, but rather to prevent ourselves from being overconfident in our own results.

As to the choice of this particular set of species, we have stated in the Introduction that our approach was problem-oriented in the sense that, as end-users, we were having a hard time identifying our specimens with the best taxonomic treatment at hand. The evidence in S1 Fig actually highlights the need for a comprehensive revision of the genus *Siderastrea* and the designation of whole syntypic series in order to adequately capture morphological variation across the Atlantic Ocean. Therefore, uncertainty in species identification should challenge even experienced taxonomists and yet, some consistent variation does exist, otherwise results would be statistically similar to or worse than those obtained for *Porites* (see Fig. 2). At any rate, the success of our approach with respect to other species certainly deserves further testing by the community at large, thus we strongly believe this manuscript to be worthy of publication.

We have also offered two additional contributions, which may be employed independently of each other: the first is the introduction of texture descriptors as means of quantifying SEMs in a fast, objective and reproducible way across a broad taxonomic range. The second is the use of E-FAMs as classifiers that allow researchers to deal with fuzzy boundaries among whatever classes they decide to bin their samples into. In this paper, fuzziness was a byproduct of the taxonomic complexity inherent to quantitative characters, but it could very well arise from biological realities, such as environmental gradients, hybridization zones, metapopulations, etc.

Finally, we believe to have also succeeded in achieving the main goal of this paper by showing that fuzzy associative memories do emulate the human perception of species boundaries in the *Siderastrea* complex. When presented only with traditional characters, Θ-FAM gets “confused” (Fig. 2d) and finds specimens to have high pertinence to either species simply because these traits offer poor discrimination, making it hard do “draw lines” among them, as statistically illustrated by logistic regressions (see S12d Fig). When Θ-FAM was presented with CLBP, which approximates the visual experience of texture, “drawing the line” becomes possible because any image whose pertinence exceeded 0,70 was assigned to the “correct” species (see S12b Fig). We have thoroughly

restructured the Conclusions in an attempt to make all these contributions more evident to prospective readers.

5. “Why not include SEM images for Porites but not the main target species? Example images used during the study would also be interesting.”

R. We added sample images, please see S4 Fig.

6. “Why the same species used for CLBP were not used for the morphological analysis? This would make the method more comparable. Furthermore, a table with all morphological measurements should be provided.”

R. We did not sample morphometric characters from *Porites* because they are notoriously uninformative and the inclusion of colonies of this genus in our sampling was intended to benchmark CLBP only. We could not acquire CLBP from all *Siderastrea* spp. individuals mainly due to our limited access to the microscopy facility (some important deadlines would not be met if we tried to SEM all specimens). The lack of CLBP sampling from all colonies actually makes our hypothesis testing more conservative: because ANN performance tends to increase with the amount of training, the fact that more images were analyzed for morphometric characters than they were for CLBP should theoretically benefit the performance of Θ-FAM in the former case.

7. “Line 4: Define SI”

R. Done

8. “Lines 27-30: I can see the overlap between S. siderea from Panama and S. stellata from Paraíba on the distance among columella and the number of septa, but for the corallite diameter there is an overlap between S. siderea from Panama and S. stellata from Gulf of Mexico.”

R. We thank the reviewer for pointing this out. The color of the bar was incorrect, Santos et al. did not sample any colonies from Panama. We have fixed the figure and now it reflects what was written in the Introduction.

9. “Line 129: Did you mean S9 Fig?”

R. Fixed, now it is S2 Fig, thank you.

10. Line 140: Why did you chose to use the word “sclerits” here? I believe that corallites might be a better fit here.

R. We meant corallites, it was fixed, thank you for your careful reading.

11. “Lines 141-144: Does this mean that you didn’t evaluate the morphology of these specimens? This does not make sense.”

R. We did evaluate their morphology: we collected morphometric data from these specimens (Table 1) and they conformed well to the ranges reported for *S. stellata* in the literature (see S3 Fig and S5 Fig). According to the taxonomic *status quo* for the genus, only *S. stellata* has ever been described for Búzios. All specimens were collected from the same site (the beach spans only a few hundred feet) and have exceptionally large sizes, further increased by colony fusion, as remarked in the

previous version of the manuscript. Búzios is subjected to upwelling, so the average temperature of sea water is lower than what is reported throughout the coast to the north of this city, which is the southernmost site in the range of Brazilian symbiotic hard corals (the only other Scleractinian species found at this site is the endemic *Mussismilia hispida*). These particularities make the occurrence of any other *Siderastrea* species at this site unlikely. Nevertheless, we do see how the statement at the end of the "Species identification" section may lead the reader astray, so we have removed it from this version.

12. "Line 177: I'm not sure what do mean with "sclerite diameter""

R. Once again, we meant corallite, thanks for pointing this out.

13. "Line 185: Do you mean Table S5? Why are the measures not provided?"

R. We have carefully revised an reordered references to figures and tables in the SI, so we believe it has been fixed in this version. Images, morphometric measurements and CLBP descriptors were uploaded to Morphobank (www.morphobank.org).

14. "Line 367: Do you mean S11?"

R. See previous response, we thank the reviewer for pointing out this issue.

15. "Table 1: Include the full definition of CLBP."

R. Done

16. "Supplementary Information Line 19: I believe the correct citation is from Milne-Edwaurds (1857)."

R. Fixed, thank you.

Reviewer #2

1. "I suggest that individual voucher numbers be included for each collected specimen in order to provide a unique and traceable identification for each sample. This will aid in organizing the data associated with each specimen and facilitate future referencing and research. Please consider inserting these voucher numbers into the document for a more comprehensive and detailed documentation of the collected samples."

R. Done, please see Table S7.

2. "A flaw here is the lack of clarity in the identification of coral species, especially those of the Siderastrea genus. Although the text mentions that the species were identified according to the most recent taxonomic treatment of the genus, it also indicates that the identification was done tentatively due to the lack of clear diagnostic features. This means that there may be uncertainty about the accuracy of Siderastrea species identifications. Additionally, the presence of specific diagnostic features for some species (such as the presence of intra-tentacular budding for S. stellata) may not be conclusive, which can lead to errors in species identification. Therefore, the flaw lies in the lack of definitive methods or diagnostic characteristics to accurately identify coral species."

7

R. The response to the 4th comment of Reviewer #1 should fully address these concerns.
